# The relationship between miR-21, DNA methylation, and bisphenol a in bovine COCs and granulosa cells

**DOI:** 10.3389/fcell.2023.1294541

**Published:** 2023-11-15

**Authors:** Reem Sabry, Diana R. May, Laura A. Favetta

**Affiliations:** Reproductive Health and Biotechnology Laboratory, Department of Biomedical Sciences Ontario Veterinary College, University of Guelph, Guelph, ON, Canada

**Keywords:** miR-21, DNA methylation, bisphenol A, oocytes, granulosa cells

## Abstract

**Introduction:** miR-21 is a critical microRNA for the regulation of various processes in oocytes and granulosa cells. It is involved in the modulation of apoptosis and can influence other epigenetic mechanisms. Among these mechanisms, DNA methylation holds significant importance, particularly during female gametogenesis. Evidence has demonstrated that microRNAs, including miR-21, can regulate DNA methylation. Bisphenol A (BPA) is a widespread chemical that disrupts oocyte maturation and granulosa cell function. Recent findings suggested that BPA can act through epigenetic pathways, including DNA methylation and microRNAs.

**Methods:** This study uses anti-miR-21 LNAs to explore the involvement of miR-21 in the regulation of DNA methylation in bovine Cumulus-Oocyte-Complexes (COCs) and granulosa cells, in the presence and absence of BPA. This study investigated 5 mC/5hmC levels as well as gene expression of various methylation enzymes using qPCR and western blotting.

**Results and discussion:** Results reveal that BPA reduces 5mC levels in granulosa cells but not in COCs, which can be attributed to a decrease in the methylating enzymes DNMT1 and DNMT3A, and an increase in the demethylating enzyme TET2. We observed a significant increase in the protein levels of DNMT1, DNMT3A, and TET2 upon inhibition of miR-21 in both COCs and granulosa cells. These findings directly imply a strong correlation between miR-21 signaling and the regulation of DNA methylation in bovine COCs and granulosa cells under BPA exposure.

## 1 Introduction

Oocyte competence, essential for embryo development, includes equipping the oocyte with the critical RNA, proteins, and nutrients required for appropriate embryo development (Coticchio et al., 2015). Whether an oocyte is developmentally competent, is heavily dependent on intricate molecular pathways governed by both genetic and epigenetic mechanisms. Epigenetics, the study of molecular pathways capable of altering gene expression without altering the DNA sequence, consists of DNA methylation, histone modifications, and non-coding RNA (ncRNA) (Felsenfeld, 2014). The disruption of any of these pathways during the critical window of oocyte maturation can have detrimental effects on subsequent fertilization, early embryo development, and the birth of a live offspring (Jacobs et al., 2017). The endocrine disrupting compound bisphenol A (BPA) is capable of interfering with all three epigenetic pathways (Jacobs et al., 2017). It is shown that these pathways govern thousands of regulatory processes essential for adequate cellular function. There is also increasing evidence to suggest that these pathways can control each other in a complex regulatory network that remains to be uncovered.

The focus of this study is on a specific microRNA, miR-21, and its ability to orchestrate DNA methylation in bovine COCs and granulosa cells. The highly complex nature of miRNA synthesis indicates that multiple routes of disruption are possible. Estrogen and androgen signaling are examples of hormonal pathways that are involved in regulating miRNA expression in different tissues (Gulyaeva and Kushlinskiy, 2016). Environmental factors including endocrine-disrupting compounds, such as bisphenols, are capable of interfering with gene expression via miRNA activity (Huumonen et al., 2014; Gulyaeva and Kushlinskiy, 2016).

miR-21 is an important miRNA conserved in several species such as rats, bovine, and humans (Yerushalmi et al., 2018). It is critical for mammalian reproduction and abundantly expressed in bovine, rats, pigs, and human oocytes and granulosa cells (Yerushalmi et al., 2018). Previous research by our group reports that BPA significantly increases miR-21 expression in bovine granulosa cells and Cumulus-Oocyte-Complexes (COCs) (Sabry et al., 2021). miR-21 is important for cell survival by regulating apoptotic genes such as PDCD4, PRC1, and CDC25a (Zi et al., 2017; Yerushalmi et al., 2018). miR-21 knockdown studies in pigs and mice report disrupted meiotic maturation and increased apoptosis of cumulus cells and embryonic arrest at the 4 - 8 cell stage due to the inhibition (Han et al., 2017). The DNA region that encodes miR-21 contains an estrogen response element (ER) in its promoter; therefore, it has the potential to be regulated by estrogen and estrogen-mimic compounds, such as bisphenols (Klinge, 2009).

Another crucial epigenetic pathway is DNA methylation, which involves the addition by specialized enzymes of a methyl group on specific sites within the genome, known as CpG islands (Jin et al., 2011). These methyl marks most commonly repress transcription and thereby govern which genes are turned “on or off” in which cells and during which developmental stage. DNA methylation functions to maintain genomic integrity, imprint on specific genes, and inactivate the X chromosome (Fedoriw et al., 2012; Toranõ et al., 2016). DNA methyltransferases (DNMTs) are responsible for the methyl addition within the genome to either maintain methylation (DNMT1) or to establish *de novo* methylation patterns (DNMT3s). Other genes involved in DNA demethylation, such as TETs and TDG, also play critical roles in regulating genome wide demethylation to activate gene expression. Decreased levels of DNMT3 enzymes are linked to embryonic lethality (Liao et al., 2015), several cancers (Ramassone et al., 2018), and EDC exposure, including BPA (Chao et al., 2012). Similar to miRNAs, methylation pathways and DNMT activities can also be affected by BPA. In development, BPA exposure resulted in abnormal methylation on the X chromosome, decreased DNMT activity, inhibition of meiotic development, and genome-wide methylation errors (Chao et al., 2012; Kim et al., 2013; Trapphoff et al., 2013; Kahlon, 2016; Wang et al., 2016). Previous research by our group reported that BPA exposure was correlated with significant increases and decreases in DNMT3A mRNA and protein expression, respectively, in bovine granulosa cells (Sabry, 2019).

Recently, researchers have begun to investigate the interactions between miRNAs and DNA methylation: an intricate regulatory network exists between these epigenetic modulators to control gene expression. By altering the promoter regions of miRNA genes on the genome, DNA methylation can modulate the biosynthesis of miRNAs (Lujambio et al., 2008). miR-34b and miR-34c, which are tumour suppressor miRNAs that target a number of oncogenes, are tightly regulated by the methylation status of their primary gene promoter region in cancer cells (Suzuki et al., 2010).

On the other hand, miRNAs can regulate DNA methylation by targeting and modulating genes that encode for methylation enzymes and other factors that comprise the DNA methylation machinery. miR-29b is found to target DNMT3A and 3B, thereby regulating *de novo* methylation of several target genes (Fabbri et al., 2007). Furthermore, miR-148a has been shown to target DNMT1, responsible for the maintenance DNA methylation (Shimizu et al., 2020). Additionally, miR-21 was shown to be able to modulate the expression of TET2, which is responsible for the demethylation of DNA (Cao et al., 2019). Furthermore, miR-21 can regulate the expression of DNMT3A, and thereby control the activation of the promoter regions that promote hepatocellular carcinoma (HCC) cell growth and proliferation (Lin et al., 2023). Additionally, it was reported that treatment with a miR-21 inhibitor increased DNMT3A expression, increased promoter methylation patterns, and arrested the growth of these HCC cells (Lin et al., 2023).

A significant increase and decrease of miR-21 expression and the methylation regulator, DNA methyltransferase 3A (DNMT3A), respectively, after BPA treatment suggests a plausible link between these two genes in the context of female reproductive toxicity. DNMT3A is one of the predicted downstream targets of miR-21 in *in vitro* bovine embryos (Mondou et al., 2012). These studies present interesting correlations suggesting miR-21 regulation of DNA methylation. Knocking down miR-21 in bovine cumulus cells using locked nucleic acid (LNA) technology is a simple, precise, and accurate method to answer whether this interplay between epigenetic regulators exists in bovine oocytes and granulosa cells during *in vitro* oocyte maturation and *in vitro* granulosa cell culture. Any alteration to DNMT levels most likely contributes to downstream disruptions of maintenance and *de novo* DNA methylation of crucial genes within the oocyte, which leads to altered gene expression, and ultimately disrupted oocyte maturation.

miRNAs and DNA methylation are essential epigenetic mechanisms that regulate gene expression in various cell types. These vital mechanisms interact through complex regulatory processes co-orchestrating the precise tuning of gene expression and cellular functions. Their interaction carries severe biological implications on a broad range of physiological operations, including development, differentiation, and disease progression. This research is focused on exploring miR-21’s functional capacity to regulate the DNA methylation process. By analyzing their intertwined partnership, we aim to obtain insights into the convoluted regulatory mechanisms governing these cellular functions in both healthy and environmentally-disrupted reproductive functions.

## 2 Results

### 2.1 miR-21 was inhibited with an 80% efficiency in bovine COCs after transfection with anti-miR-21 LNAs

**FIGURE 1:**
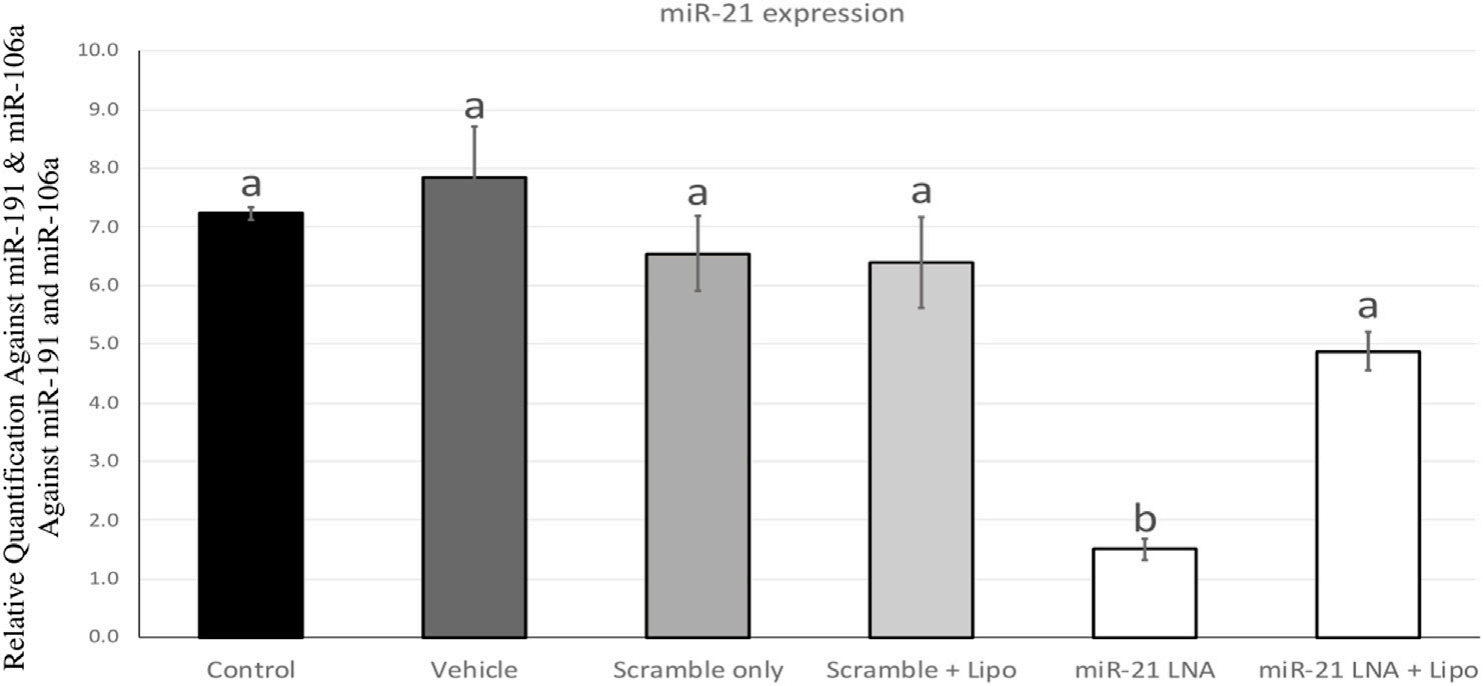
miR-21 expression in *in vitro* matured bovine COCs. Bovine COCs were matured in *in vitro* S-IVM media and treated with either an LNA scramble or LNA inhibitor in the presence and absence of Lipofectamine 3,000 at 0.1 µM and 0.5 µM, respectively. Results show that treatment with the miR-21 inhibitor alone resulted in an 80% knockdown of miR-21 expression. Different letters indicate significant differences, with *b* indicating a significantly different mean than *a* at *p* < 0.05. Bars represent the mean ± SEM.

miR-21 was inhibited in bovine COCs over the course of *in vitro* maturation. The transfection and knockdown efficiencies were previously confirmed in *in vitro* cultured granulosa cells (Sabry et al., 2022) and therefore, miR-21 inhibition will be confirmed only in COCs. Figure 1 depicts miR-21 expression in bovine COCs with both the scramble and inhibitor LNAs in the presence and absence of Lipofectamine 3,000. The results show a significant reduction in miR-21 expression when the inhibitor was used alone at 0.5 µM with no changes in the scramble group at that same concentration [F (5,12) = 16.7, *p* = 0.0000486477]. The difference between the control and the miR-21 LNA-only groups is an 80% (±1.8%) reduction in expression values indicating effective inhibition of miR-21 activity. This concentration was used for further transfection of COCs followed by treatments with either ethanol alone or ethanol with BPA at 0.05 mg/mL.

**FIGURE 2:**
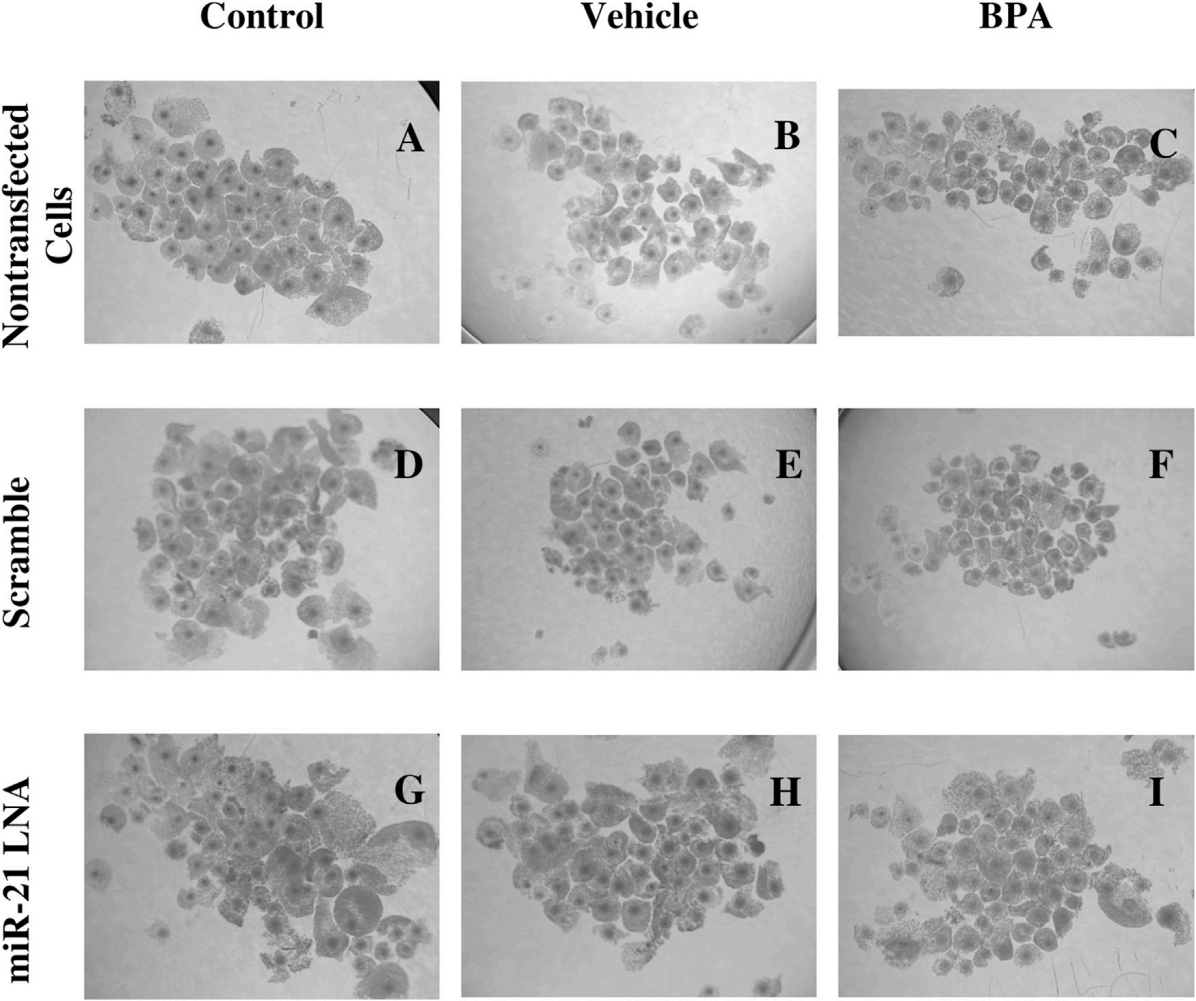
Oocyte Morphology after 24 h *in vitro* maturation with anti-miR-21 LNAs and BPA. Bovine COCs were placed into maturation media alone **(A–C)**, in the presence of a scramble **(D–F)**, or in the presence of anti-miR-21 LNA inhibitors **(G–I)** at 0.5 µM. 12 h into maturation, COCs were also treated with either a vehicle **(B,E,H)** or BPA **(C,F,I)** at 0.05 mg/mL for another 12 h. BPA-treated COCs appear poorer in quality with less cumulus cell expansion and increased heterogeneity among COC phenotypes. Scale bar = 500 µm.

24 h after *in vitro* maturation, the COCs from all 9 groups were imaged and are represented in Figure 2. As shown through qualitative assessment, treatment of COCs with BPA halfway through maturation negatively impacts the observable qualities of the COCs. The BPA-treated COCs exhibited incomplete cumulus expansion and the cytoplasm of the cumulus cells appeared darker and more fragmented than the control. This appeared to be the case for all BPA-treated COCs regardless of transfection conditions indicating miR-21 inhibition has no observable effect on COC phenotypes.

### 2.2 BPA significantly decreases 5 mC staining in bovine GCs

**FIGURE 3:**
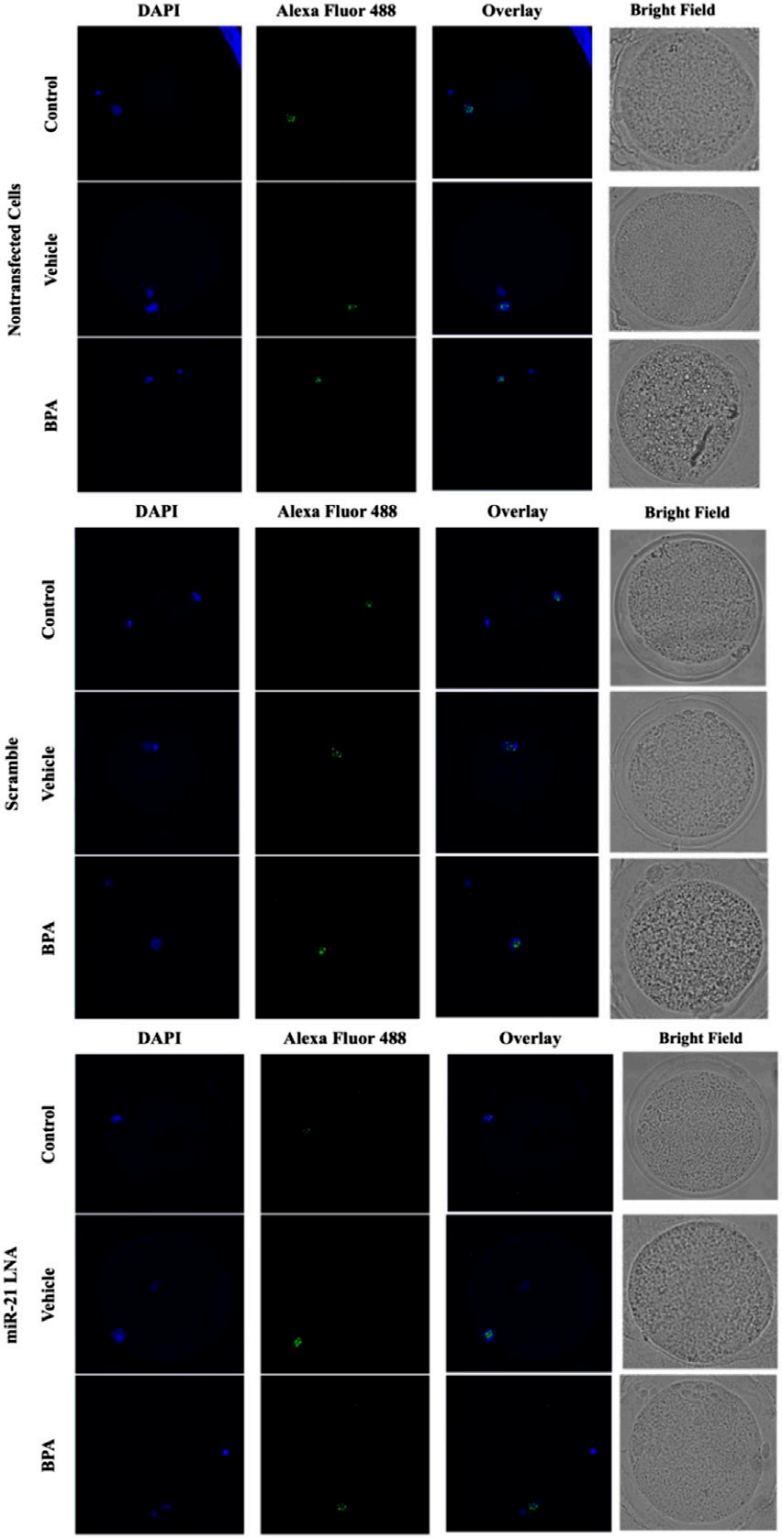
Confocal Microscopy of 5′methylcytosine in Bovine Oocytes. Transfected and treated COCs were fixed and permeabilized to allow for immunostaining of 5 mC. Immunodetection of 5 mC in COCs was achieved by utilizing an Alexa Fluor 488 conjugated secondary antibody coupled with confocal microscopy. Confocal Images are represented for all 9 groups.

**FIGURE 4:**
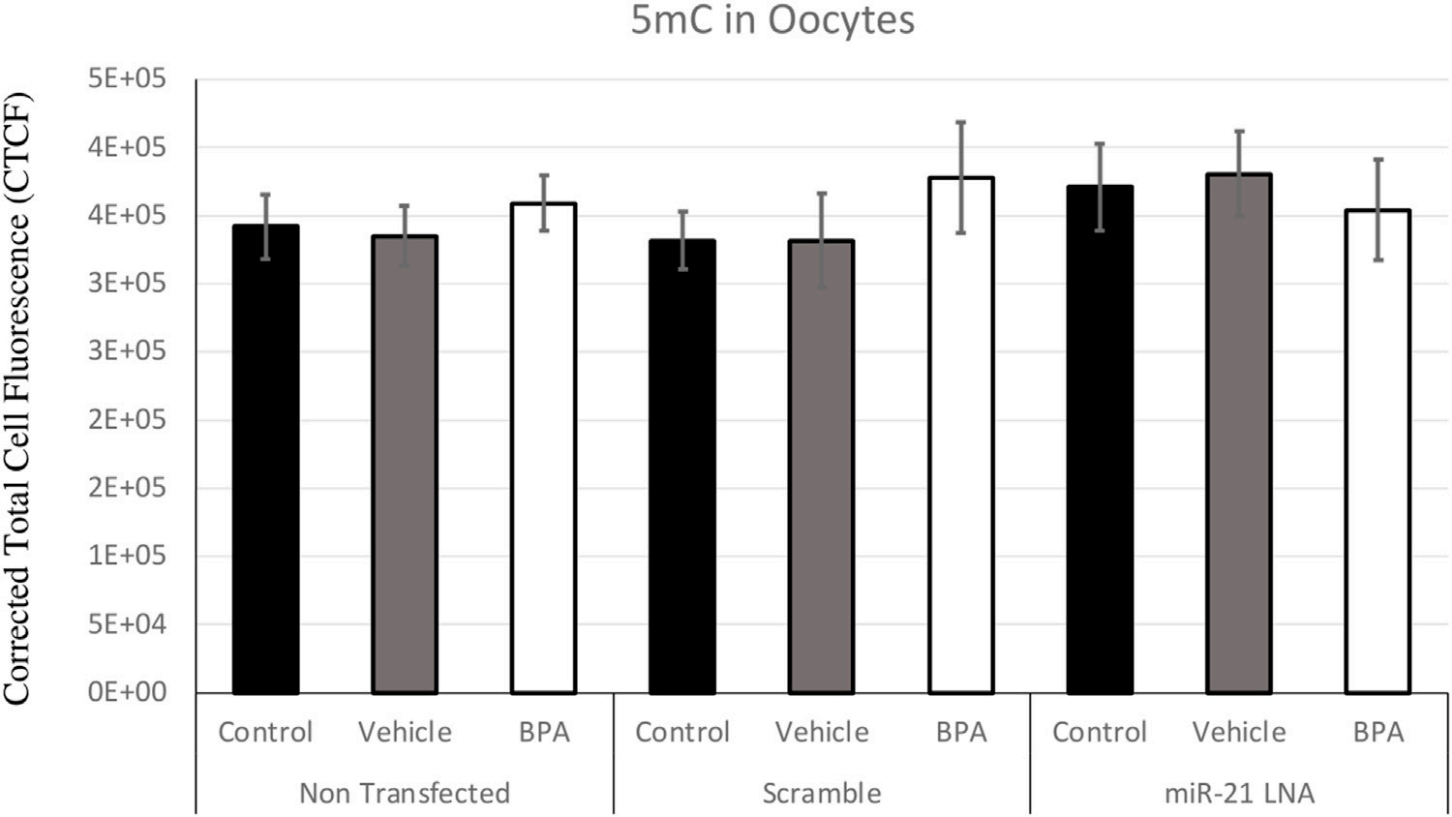
Corrected Total Cell Fluorescence of 5′methylcytosine in Bovine Oocytes. Transfected and treated COCs were fixed and permeabilized to allow for immunostaining of 5 mC. The corrected total cell fluorescence was calculated using ImageJ on a minimum of 15 COCs per group for a total of 140 COCs analyzed. Bars represent the mean ± SEM at *p* < 0.05.

**FIGURE 5:**
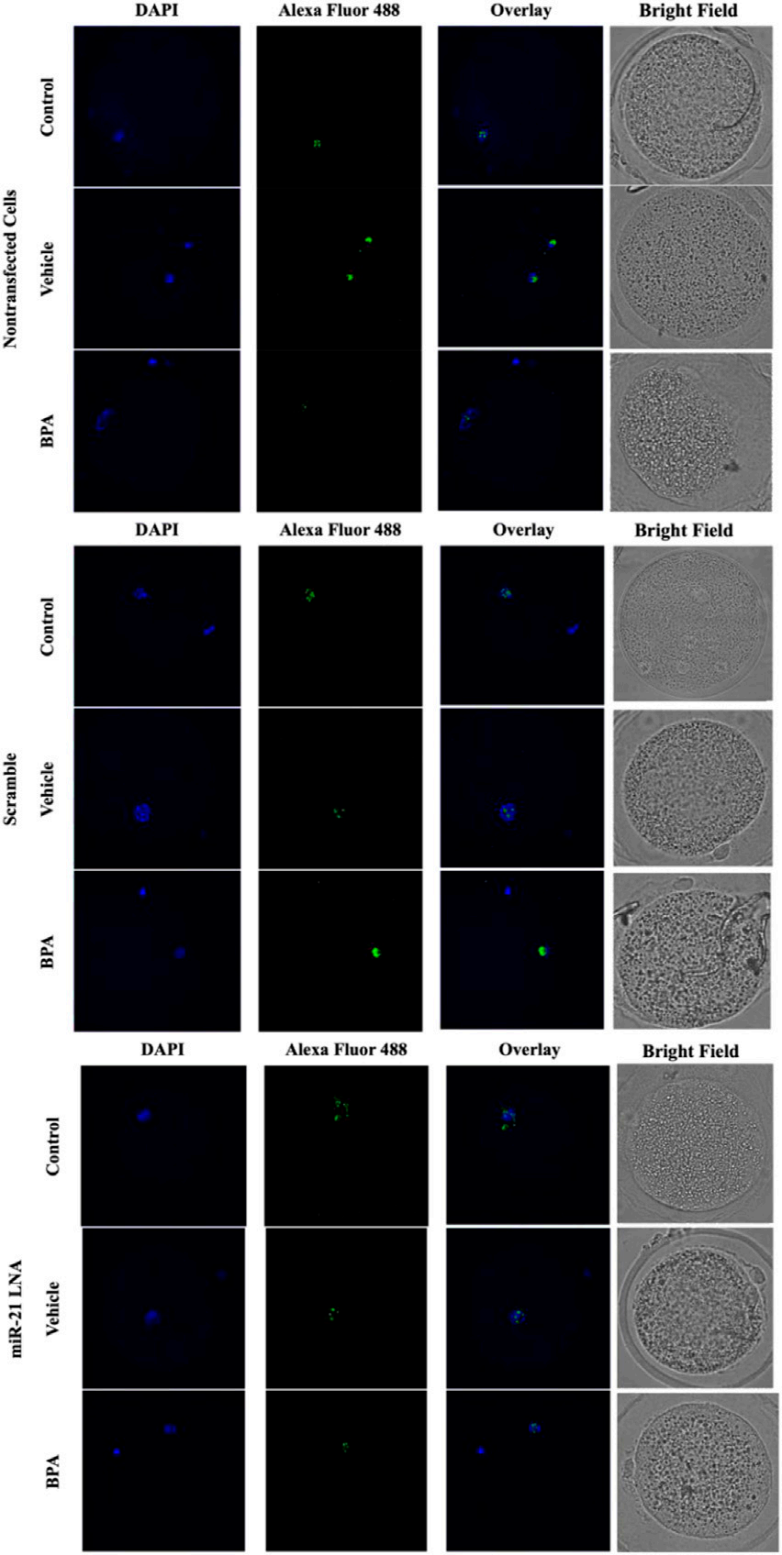
Confocal Microscopy of 5′hydroxymethylcytosine in Bovine Oocytes. Transfected and treated COCs were fixed and permeabilized to allow for immunostaining of 5 hmC. Immunodetection of 5 hmC in COCs was also achieved by utilizing an Alexa Fluor 488 conjugated secondary antibody coupled with confocal microscopy. Confocal Images are represented for all 9 groups.

**FIGURE 6:**
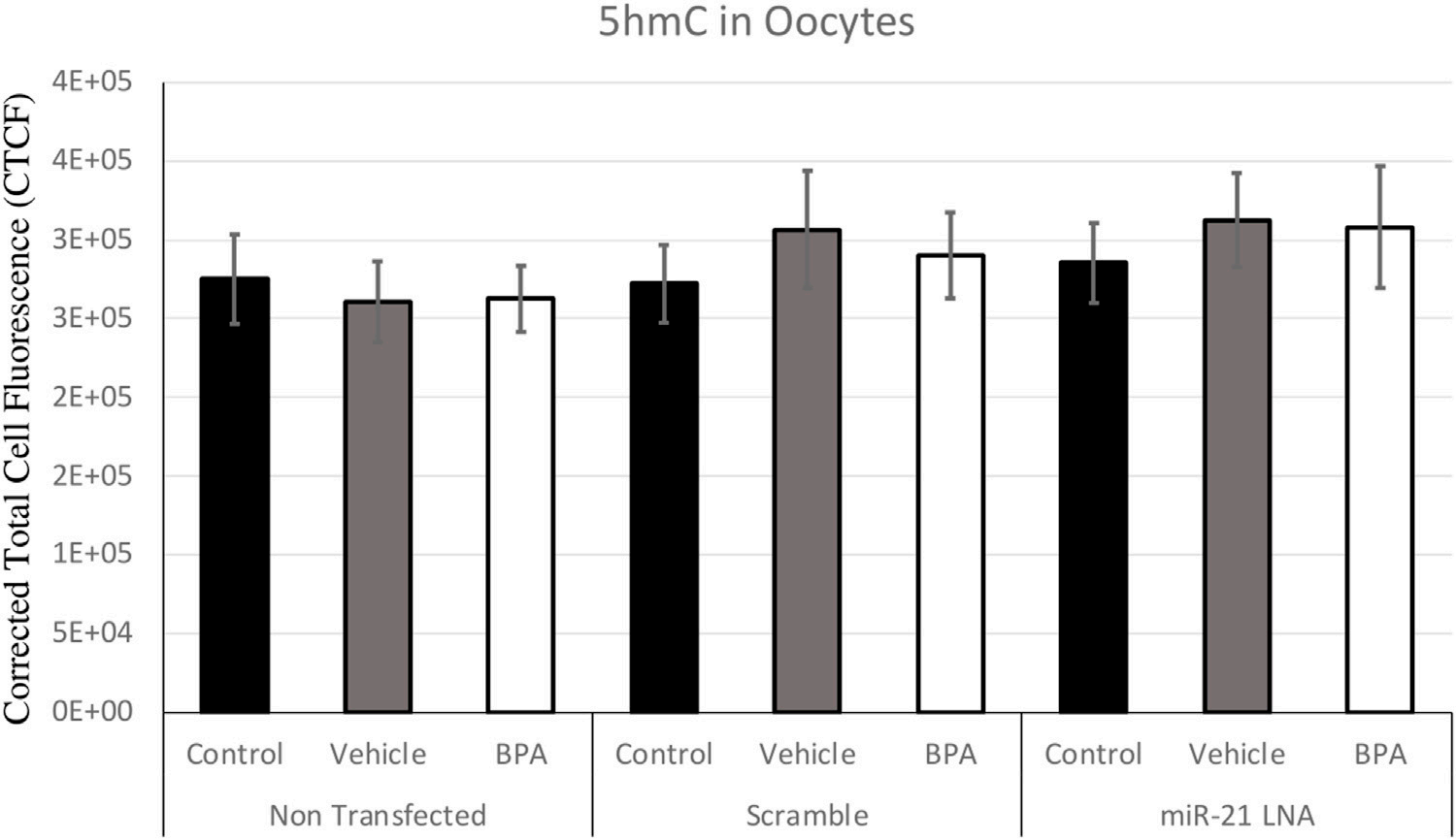
Corrected Total Cell Fluorescence of 5′hydroxymethylcytosine in Bovine Oocytes. Transfected and treated COCs were fixed and permeabilized to allow for immunostaining of 5 hmC. The corrected total cell fluorescence was calculated using ImageJ on a minimum of 15 COCs per group for a total of 140 COCs analyzed. Bars represent the mean ± SEM at *p* < 0.05.

Immunofluorescence of 5 mC and 5 hmC provided initial information on the effects of BPA on global methylation patterns and whether these effects are reliant on miR-21-mediated pathways. Figures 3–6 display the results of 5 mC and 5 hmC staining in oocytes, which displayed that BPA had no significant effects on global methylation patterns as shown by confocal microscopy (Figures 3, 5) and analyzed by ImageJ (Figures 4, 6).

**FIGURE 7:**
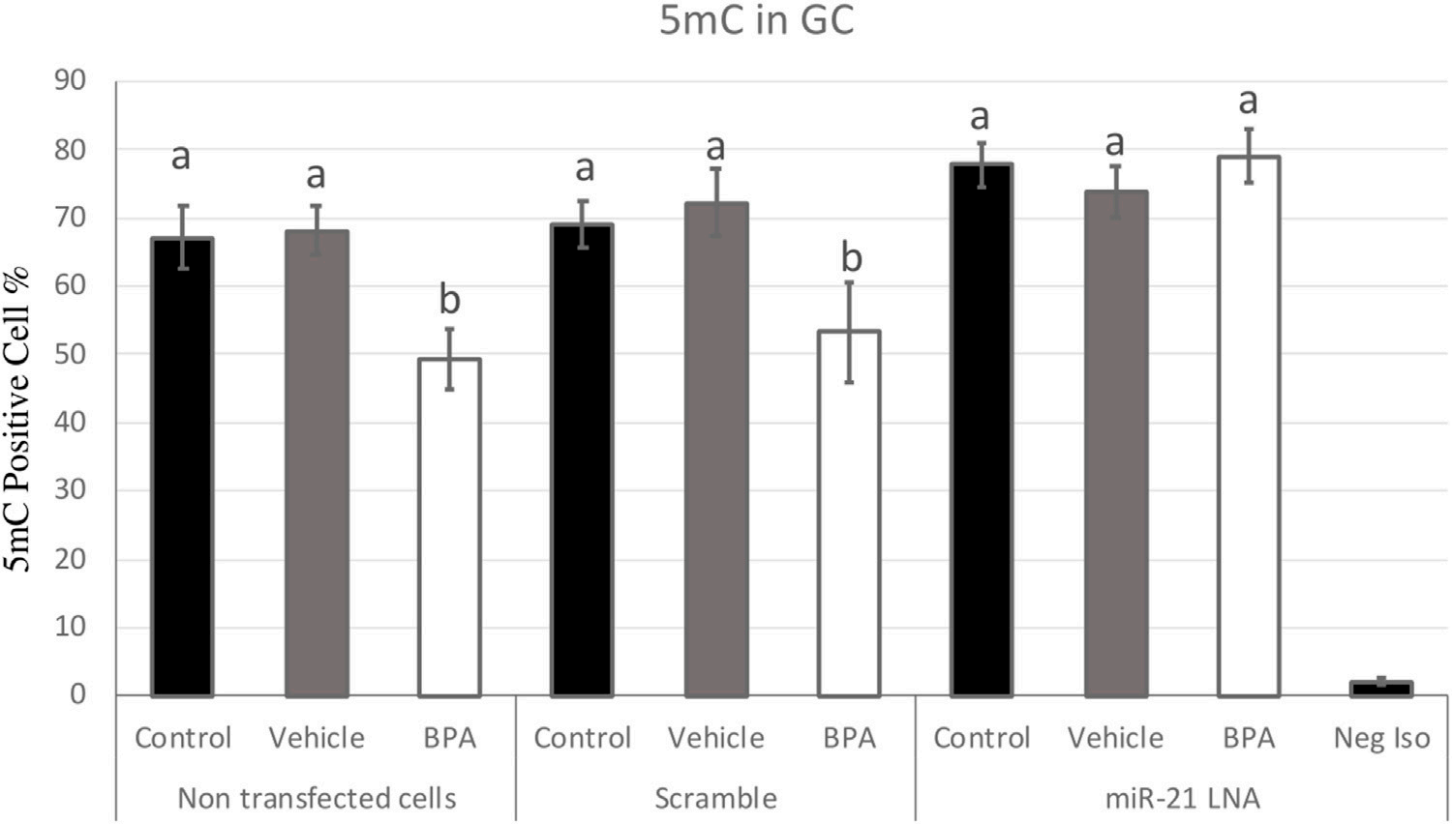
Quantification of 5′methylcytosine (5 mC) in Bovine Granulosa Cells. Analysis of 5 mC levels was done on FlowJo on a minimum of three biological replicates. The last column represents the negative control where a negative isotype was used as the primary antibody. Different letters indicate significant differences, with *b* indicating a significantly different mean than *a* at *p* < 0.05. Bars represent the mean ± SEM and *n* = 7.

**FIGURE 8:**
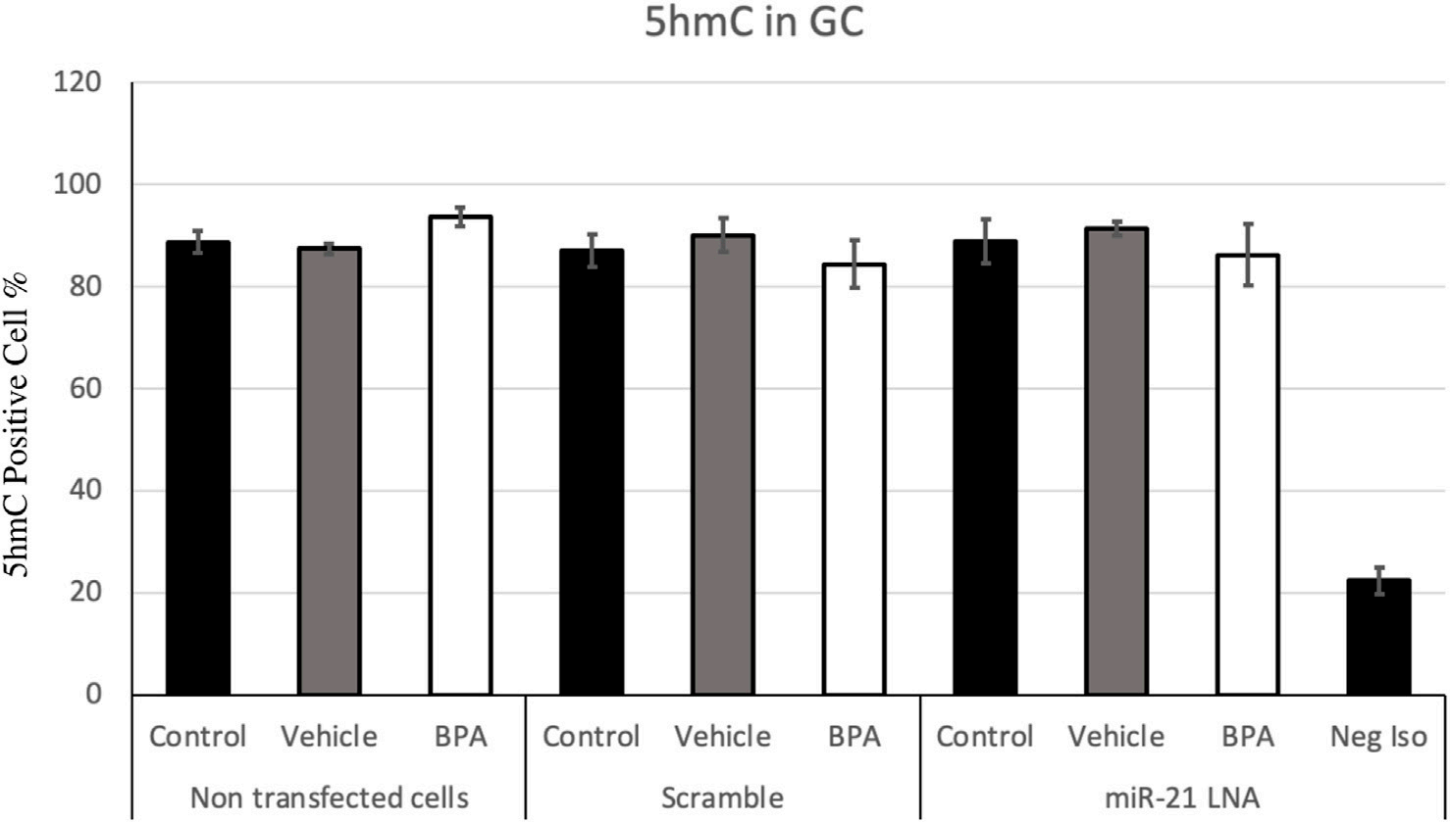
Quantification of 5′hydroxymethylcytosine (5 hmC) in Bovine Granulosa Cells. The analysis was done on FlowJo on a minimum of three biological replicates. The last column represents the negative control where a negative isotype was used as the primary antibody. Bars represent the mean ± SEM at *p* < 0.05and *n* = 4.

Figure 7, 8 display the results of 5 mC and 5 hmC staining in GCs, which interestingly revealed opposite findings than the COCs. BPA significantly decreased 5 mC staining in GCs as shown by Flow cytometry (Supplementary Figure S2). Analysis using FlowJo showed that BPA reduces 5 mC staining in nontransfected GCs and in scramble GCs [H (8) = 23.4562, *p* = 0.002825] (Figure 7). Additionally, miR-21 inhibition in GCs reversed the effects of BPA on 5 mC staining in GCs where there are no significant changes between the inhibited controls and treated groups (Figure 7). Similar to the COCs, miR-21 inhibition and BPA did not alter 5 hmC staining in GCs either (Figure 8).

### 2.3 BPA alters the gene expression profile of both methylation and demethylation genes in COCs and GCs

**FIGURE 9:**
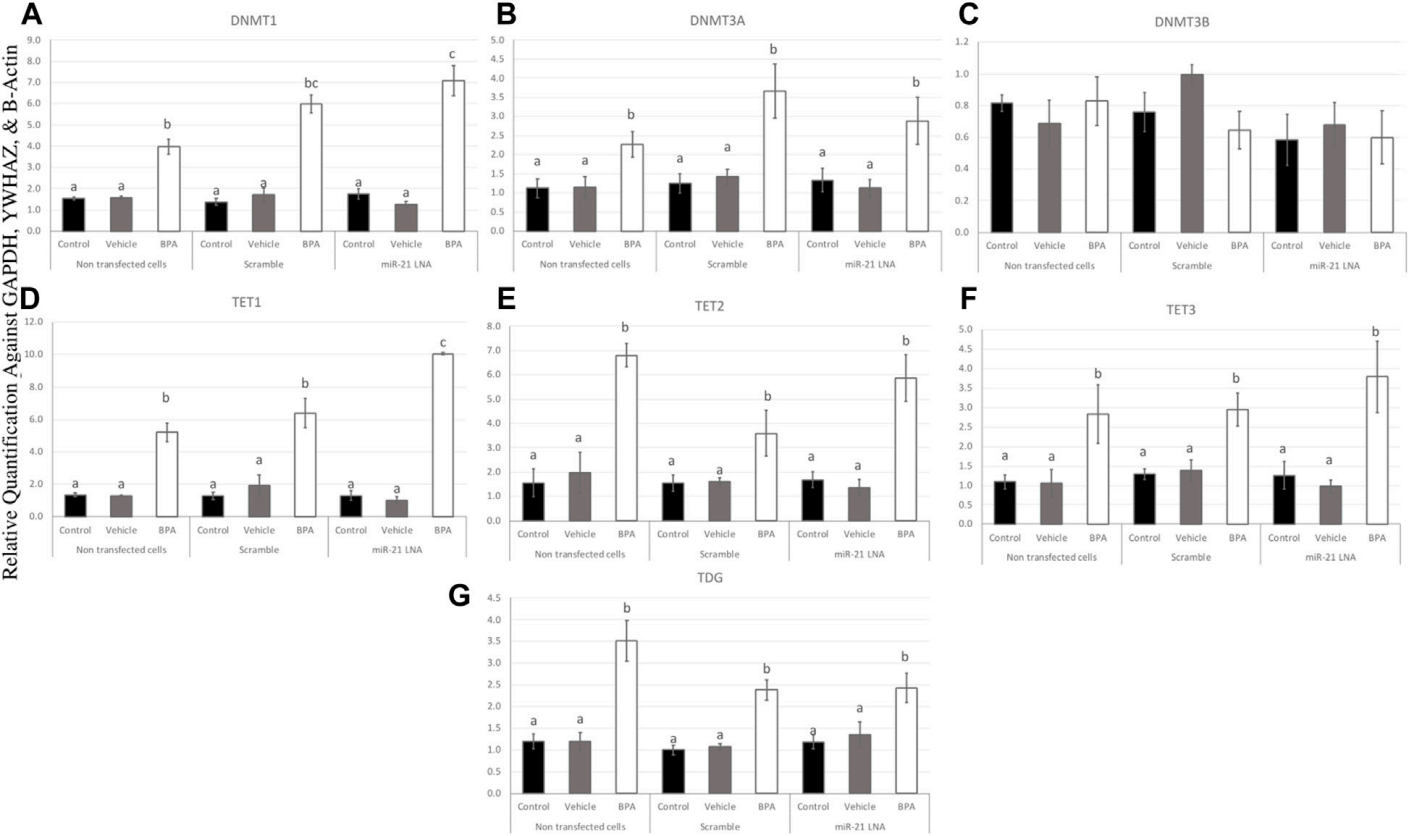
Expression of methylating and demethylating mRNAs after miR-21 inhibition and BPA treatment in Bovine COCs. COCs were transfected with LNA inhibitor probes at 0.5 μM then treated with BPA (0.05 mg/mL). All transcripts including DNMT1 **(A)**, DNMT3A **(B)**, TET1—3 **(D–F)**, and TDG **(G)** were significantly increased after BPA exposure in all transfection groups apart from DNMT3B **(C)**. Quantification is normalized to reference targets GAPDH, YWHAZ, and B-Actin. Different letters indicate significant differences, with *b* indicating a significantly different mean than *a* at *p* < 0.05, *c* indicating a significantly different mean than *a* and *b*, and *d* indicating a significantly different mean than *a*, *b*, and *c* at *p* < 0.05. *ab* indicates no differences between *a* or *b* and *bc* indicates no differences between *b* or *c*. Bars represent the mean ± SEM.

**FIGURE 10:**
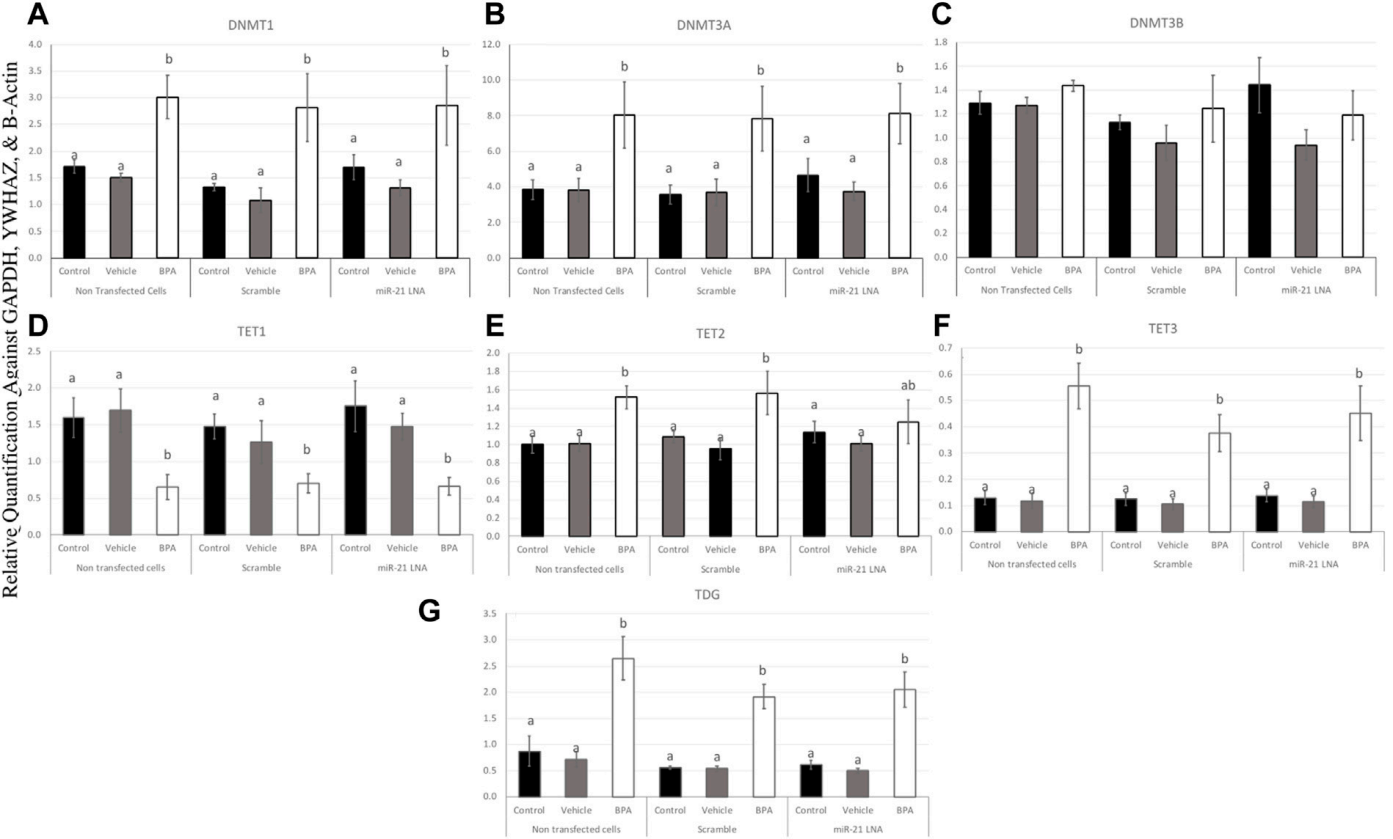
Expression of methylating and demethylating mRNAs after miR-21 inhibition and BPA treatment in Bovine GCs. Cells were transfected with LNA inhibitor probes at 0.5 μM for 12 h and then treated with BPA (0.05 mg/mL) for another 12 h. Most transcripts including DNMT1 **(A)**, DNMT3A **(B)**, TET3 **(F)**, and TDG **(G)** were significantly increased after BPA exposure in all transfection groups apart from TET1 **(D)** that was decreased, DNMT3B **(C)** that was unaffected, and TET2 **(E)** that was unaffected in the inhibited group. Quantification is normalized to reference targets GAPDH, YWHAZ, and B-Actin. Different letters indicate significant differences, with *b* indicating a significantly different mean than *a* at *p* < 0.05. Bars represent the mean ± SEM.

To further uncover the source of aberrant methylation patterns due to BPA exposure, the expression of methylation and demethylation transcripts were quantified using qPCR. Methylators (DNMT1, 3A, and 3B) and demethylators (TET1, 2, 3, and TDG) were quantified and normalized to housekeeping genes (GAPDH, B-actin, and YWHAZ). Figures 9, 10 display the results in COCs and GCs, respectively. In COCs, all transcripts except for DNMT3B were significantly increased after BPA exposure regardless of transfection conditions (*p* < 0.05) (Figure 9). In GCs, the results are similar to the COCs for the methylators investigated; DNMT1 and DNMT3A were significantly increased after BPA exposure, while DNMT3B was unaffected (Figures 10A–C).

In terms of the demethylators, TET3 and TDG were also increased in the BPA-treated cells regardless of transfection conditions (*p* < 0.05) (Figures 10F, G). TET2 was also significantly increased after BPA treatment, but only in the nontransfected and scramble groups (*p* < 0.05), whereas the miR-21 inhibited cells that were also treated with BPA exhibited a non-significant increase in TET2 (Figure 10E). Finally, TET1 was the only transcript in GCs that was significantly decreased as a result of BPA exposure and this was independent of miR-21 inhibition (*p* < 0.05) (Figure 10D). This was the opposite of the significant increase of this gene observed in BPA-treated COCs (Figure 9D). The individual statistic H, F, and *p* values for each target can be found in Supplementary Table S2.

### 2.4 miR-21 inhibition increases the expression of DNMT1 and TET2 protein but not DNMT3A in COCs

**FIGURE 11:**
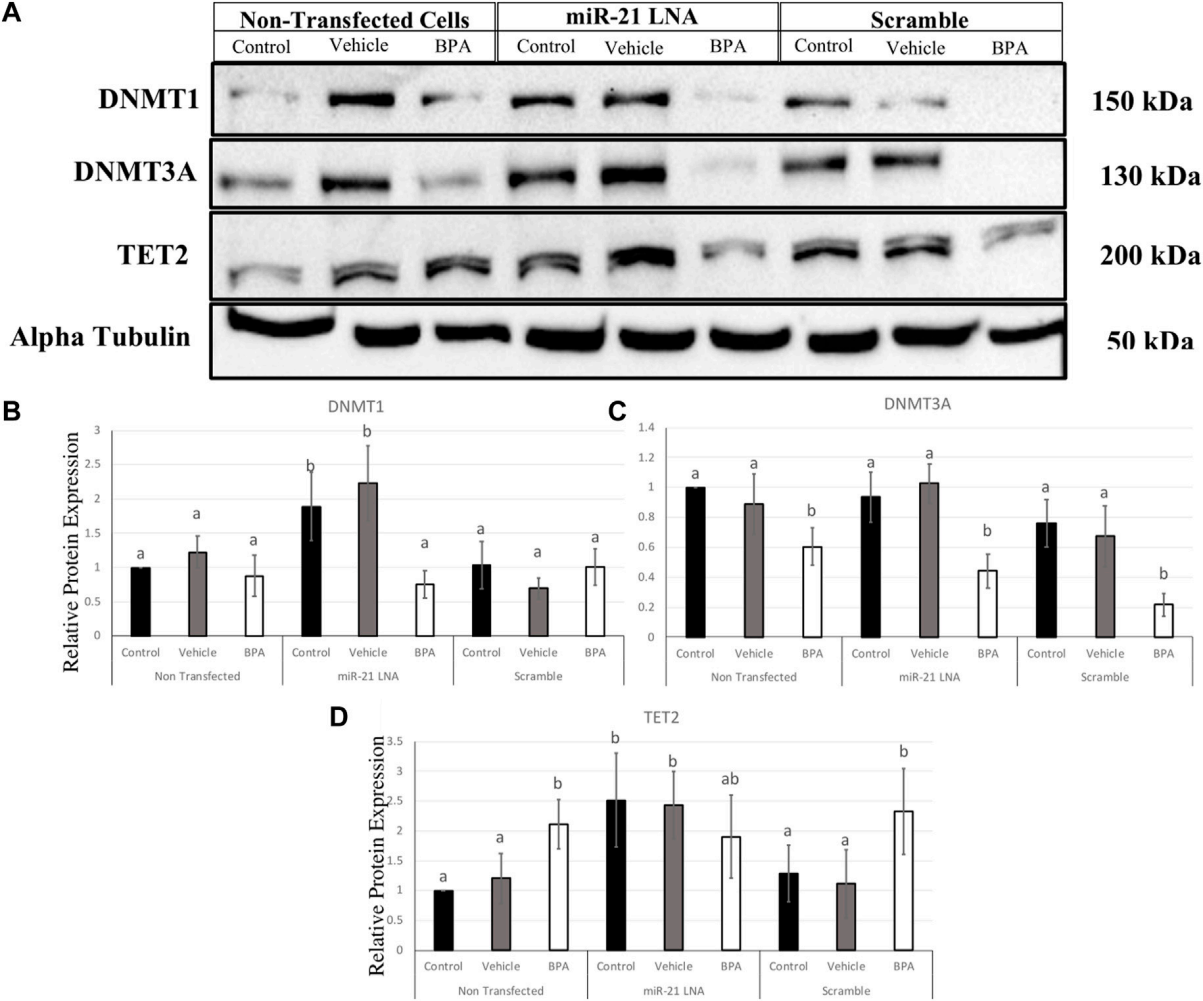
Relative protein expression of DNMT1, DNMT3A, and TET2 after miR-21 inhibition and BPA treatment in Bovine COCs. Transfections were done with LNA inhibitor probes at 0.5 μM for 12 h followed by BPA treatment for another 12 h. Western blots **(A)** and graphical representations of DNMT1 **(B)**, DNMT3A **(C)**, and TET2 **(D)** revealed that BPA decreased and increased DNMT3A and TET2 proteins, respectively. miR-21 Inhibition also induced protein expression in the control-only groups for DNMT1 and TET2. Densitometric analysis was performed relative to the loading control, α-tubulin. Different letters indicate significant differences, with *b* indicating a significantly different mean than *a* at *p* < 0.05. Bars represent the mean ± SEM. *n* = 6 for DNMT1; *n* = 9 for DNMT3A; *n* = 6 for TET2.

To interpret the effects of miR-21 inhibition and BPA on key methylation genes, it is important to quantify the protein levels to demonstrate if these effects are translating. In COCs, DNMT1, DNMT3A, and TET2 were quantified at the protein level (Figure 11A). DNMT1 was unaffected by BPA but interestingly, miR-21 inhibition alone significantly increased DNMT1 protein expression in the control groups (*p* = 0.021) and in the vehicle groups (*p* = 0.005) (Figure 11B). DNMT3A, on the other hand, was affected by BPA exposure but not by miR-21 inhibition. DNMT3A was significantly decreased after BPA exposure in the nontransfected group (*p* = 0.046), in the scramble group (*p* = 0.011), and in the inhibited group (*p* = 0.014). Unlike DNMT1, there is no significant increase of DNMT3A after miR-21 inhibition (Figure 11C).

BPA had the opposite effect on TET2 protein with a significant increase in protein levels in the nontransfected group (*p* = 0.015) and in the scramble group (*p* = 0.049), but not in the inhibited group (Figure 11D), this can be attributed to a significant increase in TET2 protein in the control and vehicle groups with miR-21 inhibition. Just like the DNMTs, TET2 was shown to be significantly upregulated when miR-21 was inhibited in the control group (*p* = 0.028) and in the vehicle group (*p* = 0.048) indicating it is implicated in miR-21 signaling.

### 2.5 miR-21 inhibition increases the expression of DNMT1 and DNMT3A protein but not TET2 in GCs

**FIGURE 12:**
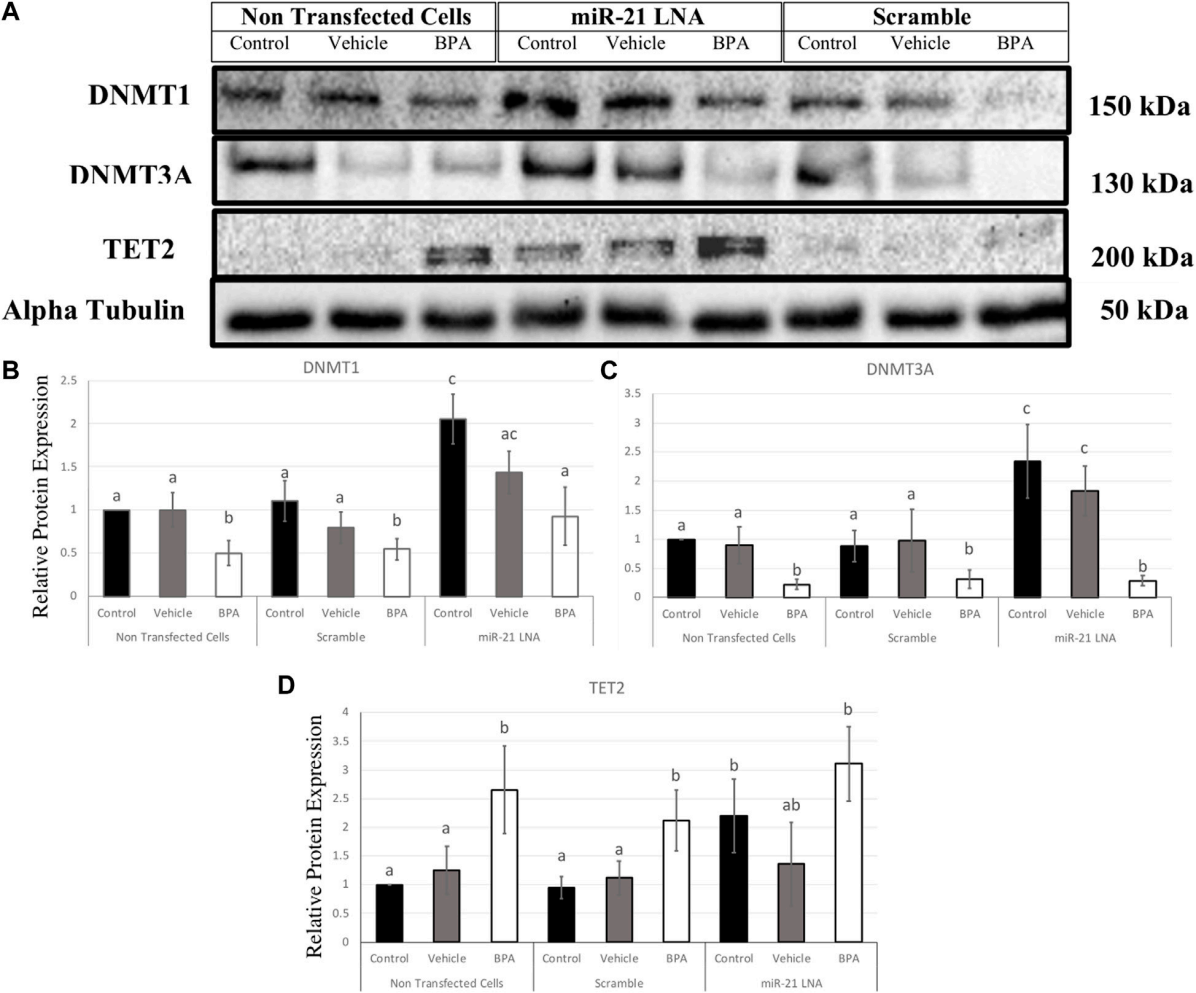
Relative protein expression of DNMT1, DNMT3A, and TET2 after miR-21 inhibition and BPA treatment in Bovine GCs. Transfections were done with LNA inhibitor probes at 0.5 μM for 12 h followed by BPA treatment for another 12 h. Western blots **(A)** and graphical representations of DNMT1 **(B)**, DNMT3A **(C)**, and TET2 **(D)** revealed that BPA decreased and increased DNMTs and TET2 proteins, respectively. miR-21 Inhibition also induced all protein expression in the control-only groups. Densitometric analysis was performed relative to the loading control, α-tubulin. Different letters indicate significant differences, with *b* indicating a significantly different mean than *a* at *p* < 0.05. Bars represent the mean ± SEM. *n* = 9 for DNMT1, *n* = 6 for DNMT3A, *n* = 6 for TET2.

In GCs, DNMT1, DNMT3A, and TET2 were also quantified at the protein level (Figure 12A). DNMT1 was significantly decreased after BPA exposure in the nontransfected group (*p* = 0.049), in the scramble group (*p* = 0.049), and in the inhibited group (*p* = 0.038) (Figure 12B). Interestingly, miR-21 inhibition alone significantly increased DNMT1 protein expression in the control groups (*p* = 0.004), the vehicle groups (*p* = 0.048), and there was a significant recovery of DNMT1 after BPA exposure in the miR-21 inhibited group (*p* = 0.024) (Figure 12B).

Similarly, DNMT3A exhibited similar results in terms of BPA exposure and miR-21 inhibition. DNMT3A was significantly decreased after BPA exposure in the nontransfected group (*p* = 0.025), in the scramble group (*p* = 0.02), and in the inhibited group (*p* = 0.005). Furthermore, miR-21 inhibition alone significantly increased DNMT3A protein expression, just like DNMT1, in the control groups (*p* = 0.011) and in the vehicle groups (*p* = 0.039). Unlike DNMT1, there is no significant recovery of DNMT3A after BPA exposure in the miR-21-inhibited group (Figure 12C).

BPA had the opposite effect on TET2 protein with a significant increase in protein levels regardless of transfection conditions. This was seen in the nontransfected group (*p* = 0.036), in the scramble group (*p* = 0.024), and in the inhibited group (*p* = 0.029) (Figure 12D). Just like the DNMTs, TET2 was shown to be significantly upregulated when miR-21 was inhibited in the control groups (*p* = 0.037) indicating it is implicated as a miR-21 target.

## 3 Discussion

Uncovering the pathways utilized by microRNAs in oocyte maturation is key due to their crucial involvement in regulating an array of biological processes, such as cell proliferation, differentiation and apoptosis (Toms et al., 2018). In particular, research has delved into the role that individual microRNAs play in oocytes over the course of oocyte maturation; studies have demonstrated specific involvement with meiosis regulation, spindle assembly and cytoskeleton organization (Wang et al., 2017). A comprehensive analysis regarding each unique function served by these epigenetic players may provide insights into molecular mechanisms responsible for proper oocyte development, thus enhancing assisted reproductive technologies. miR-21 is one excellent example of a microRNA worth further investigations. It is upregulated during oocyte maturation in several species, including murine, porcine, bovine, and human (Tscherner et al., 2018; Dehghan et al., 2021). The functions miR-21 play in the oocyte and its surrounding granulosa cells are numerous.

miR-21 expression is crucial in granulosa cells surrounding the oocyte (Aldakheel et al., 2021). In accordance with Bartolucci et al. (Bartolucci et al., 2020), miR-21 expression is positively correlated with oocyte developmental competence; furthermore, mimicking miR-21 in granulosa cells led to improved developmental competence by way of promoting mitochondrial function while combating oxidative stress (Bartolucci et al., 2020). It is also noted that miR-21 is capable of regulating other epigenetic pathways including DNA methylation which is essential for proper oocyte maturation (Pan et al., 2010; Cao et al., 2019). It is linked to various aspects of oocyte maturations including spindle formation and maternal mRNA stability (Dehghan et al., 2021). Methylation of the granulosa cell genome has also been linked to granulosa cell function and subsequently oocyte competence. For example, adequate expression of DNMTs in granulosa cells is associated with proper methylation and normal cumulus cell expansion and steroidogenesis (Sagvekar et al., 2019).

Understanding the regulation of DNA methylation in these oocytes and granulosa cells is important since alterations in DNA methylation patterns can lead to developmental defects and infertility. This is carried out in this study where the role of miR-21 in regulating DNA methylation in bovine COCs and granulosa cells is investigated in a model of BPA toxicity.

This study showed that in bovine granulosa cells BPA induced hypomethylation, which was attenuated by inhibition of miR-21. The observation of hypomethylation is supported in literature where BPA treatment was associated with decreased 5 mC staining in various cell types including mouse (Khaghani et al., 2021) and porcine oocytes (Wang et al., 2016) that also reported differently methylated genes, decreased maturation rates, increased oxidative stress, and increased apoptosis. The importance of stable 5 mC presence in the oocyte has been proven in numerous studies. Ivanova et al. (Ivanova et al., 2020) found that 5 mC levels increased during meiotic progression, particularly in the pericentric heterochromatin regions, and that reducing 5 mC levels impaired meiotic progression and spindle assembly in pig oocytes. The study highlights that 5 mC dynamics play an important role in regulating oocyte quality in pigs.

The levels of 5 hmC in the oocyte and granulosa cells are equally as crucial for regulating gene expression for proper development (Sakashita et al., 2014). 5 hmC levels also significantly increase as oocytes grow and mature and this is linked with active transcription; this is reasonable considering the maturing oocyte requires fine tuning of gene expression for its specific unique needs (Sakashita et al., 2014). Regardless of this, there were no changes in 5 hmC levels due to BPA or miR-21 inhibition. This finding is supported in literature with no changes in 5 hmC levels due to BPA in mice blood (Kochmanski et al., 2018). However, it is worth noting that these effects are dependent on several factors including cell type, dose of BPA, and timing of exposure as other studies have reported BPA induced changes in 5 hmC levels (Senyildiz et al., 2017; Zheng et al., 2017).

The interesting finding at the level of global methylation patterns is the attenuation of BPA induced hypomethylation in the miR-21 inhibited granulosa cells. This suggests that BPA utilizes a mechanism likely dependent on miR-21 signalling. miR-21 expression is significantly increased due to BPA (Sabry et al., 2021). Therefore, increased miR-21 expression could be linked to hypomethylation of the genome. This speculation is supported by increased methylation (higher 5 mC staining) in the BPA treated granulosa cells that were also treated with a miR-21 inhibitor. To further characterize these pathways, this study investigated crucial methylation genes at the transcript and protein levels as microRNAs exert their functions by acting on transcripts and interrupting protein synthesis (Bhaskaran and Mohan, 2014).

At the mRNA level, BPA increased the amount of all transcripts analyzed except for DNMT3B in both COCs and in granulosa cells and TET1 in granulosa cells. TET1 mRNA was increased in COCs, but decreased in granulosa cells, whereas DNMT3B was unaffected. These effects occurred regardless of transfection conditions with another exception of TET2 mRNA being rescued after BPA treatment in the miR-21 inhibited granulosa cells. All these genes are known to play crucial roles in methylation within the oocyte (Sendžikaitė and Kelsey, 2019). DNMT3B, however, plays a more dominant role in early embryonic development as opposed to *de novo* methylation in the oocyte (Gao et al., 2020). DNMT3A is another *de novo* methylator for early embryogenesis, but has been proven to be critical for imprinting during oogenesis (Gao et al., 2020). This could explain why DNMT3B was the only transcript to be unaffected during oocyte maturation.

The significant disruptions of transcripts at the mRNA level indicate a disturbance in the bioavailability of methylation machinery within these cell types and this must contribute to aberrant DNA methylation as a result of BPA. These results are supported in literature with several studies showing that BPA increased the expression of DNMTs at the mRNA levels. BPA was reported to increase mRNA expression of DNMT1, TET2, and TET3 in MCF-7 cells with no changes on DNMT3A and DNMT3B (Awada et al., 2019). In addition, BPA induced an increase in DNMT1 and DNMT3A in zebrafish ovaries (Santangeli et al., 2016). These overall effects are shown to be tissue specific and dependent on experimental conditions. Bhandari et al. (Bhandari et al., 2019) reported that BPA affected DNMT mRNA expression levels differently depending on the dose of BPA used on mesenchymal cells.

Interestingly, the effects of BPA on TET2 mRNA expression in granulosa cells were reversed when miR-21 was inhibited, which suggests a link between these two genes. miR-21 has previously been shown to regulate TETs in hepatocellular carcinomas (Cao et al., 2019). They reported significant downregulation of all TET proteins when they treated cells with a miR-21 mimic (Cao et al., 2019). They suggested a direct link where miR-21 was able to directly bind to the 3′UTR sequences of TET1, TET2, and TET3. In this study, only TET2 was affected at the mRNA level in granulosa cells, supporting a novel report of miR-21 regulation of TET2 in bovine granulosa cells that are also treated with BPA. The rescue of TET2 at the mRNA level could explain the rescue that is observed in methylation patterns when miR-21 was inhibited in granulosa cells. To adequately understand the ultimate effect of BPA and miR-21 inhibition on these genes, it is crucial to quantify the functional proteins. This is also essential to confirm a functional link between miR-21 and any of these downstream methylating enzymes.

In COCs, BPA decreased and increased DNMT3A and TET2 protein levels, respectively. In granulosa cells, BPA was shown to decrease the protein levels of both DNMT1 and DNMT3A while TET2 proteins were significantly increased. A decrease in methylating enzymes and an increase in demethylating enzymes directly explain the overall hypomethylation observed in granulosa cells that were treated with BPA. The opposing finding of DNMT transcript and protein levels are not surprising since these levels are not always directly proportional (Liu et al., 2016). This also suggests the involvement of microRNAs, which are post transcriptional regulators that can interfere with protein levels without necessarily interfering with transcript levels (Bhaskaran and Mohan, 2014). The increase in transcripts can actually be a result of decreased proteins whereby the cellular response to low translation is to compensate by increasing transcription (Bhaskaran and Mohan, 2014).

In the case of TET2, the effects do appear to be directly proportional with both increased transcripts and proteins. The increase in TET2 is shown to be a direct pathway that is utilized by BPA to induce DNA hypomethylation. Li et al. (Li et al., 2020) reported that activation of the estrogen receptor by BPA modulated DNA hydroxymethylation followed by demethylation that was primarily regulated by TET2 in breast cancer cells. This effect was attenuated when TET2 was silenced (Li et al., 2020). This is supported in numerous other studies that found BPA reduced global 5 mC levels coupled with an increase in TET enzyme expression in mouse Leydig cells (Zhou et al., 2023) and mouse brains (Malloy et al., 2019).

DNMT1, DNMT3A, and TET2 were all significantly increased at the protein level in the miR-21 inhibited granulosa cells in the absence of BPA treatment. In COCs, only DNMT1 and TET2 were significantly increased as a result of miR-21 inhibition. Several recent studies characterize the ability of miRNAs to also regulate DNA methylation by targeting the transcripts for DNMTs or TETs and thereby modulating protein levels (Wang et al., 2017). miR-29b is known to target DNMTs and TETs in porcine embryos which tightly regulates DNA methylation patterns (Zhang et al., 2018). The miR-21 findings in this study are the first of its kind within this specific model; here it is reported that these three genes can be regulated by miR-21 signaling in bovine oocytes and granulosa cells. This regulation is speculated to be a direct interaction with the 3′UTR of these genes as reported in literature of other species and other cell types (Li et al., 2020).

In a study by Pan et al. (Pan et al., 2010), miR-21 and miR-148a were investigated to characterize their role in DNA hypomethylation in T cells from patients with lupus. The authors treated cells with a miR-21 mimic and reported compatible findings with significant decreases of DNMT1 at the protein level (Pan et al., 2010). As previously described, TET2 was shown to be a direct target of miR-21 in liver cancer cells (Cao et al., 2019). Furthermore, DNMT3A was predicted to be a target of miR-21 in the bovine genome as shown by Mondou et al. (Mondou et al., 2012) that investigated potential targets within the bovine embryo. Correlation analysis also revealed that miR-21 had a positive relationship with DNMT1 and DNMT3A with no significant relationship with DNMT3B in hepatocellular carcinomas (Lin et al., 2023). To the best of our knowledge, this is the first study to support these findings within bovine granulosa cells, thus rendering this relationship crucial in reproductive research. By targeting DNMT1, DNMT3A and TET2, miR-21 exhibits a significant contribution to maintaining DNA methylation patterns in bovine COCs and granulosa cells.

Despite the findings of miR-21-dependant expression of these proteins, BPA effects on these genes were not reversed when miR-21 was inhibited. DNMTs and TET2 were decreased and increased, respectively, after BPA treatment regardless of transfection conditions. This suggests that BPA effects are potent enough to mask the miR-21 regulation observed in the absence of BPA. Other microRNAs could compensate for a miR-21 knockdown and perform its function, as miRNAs are known to have high amounts of redundancies in their tasks (Fischer et al., 2015). miR-29 is another miRNA that is significantly increased after BPA treatment (Sabry et al., 2021) and it can also regulate DNMTs and TETs (Zhang et al., 2018). However, this study did observe a rescue in BPA induced hypomethylation in granulosa cells after miR-21 inhibition. Therefore, an alternative pathway by which BPA induces an increase in miR-21 expression that results in subsequent reduction of 5 mC levels in granulosa cells might exist. It is possible that there are recoveries in the other genes investigated, such as TET1 or TET3. A limitation of this study is the quantification of only TET2 at the protein level due to the commercial availability of specific antibodies that are able to detect the TET1 and TET3 isoforms within the bovine species. Antibodies for humans, mice, and rats were purchased and unsuccessfully tested on the bovine species alongside species-specific positive controls.

The discrepancies in the findings between granulosa cells and COCs can be attributed to the presence of the oocyte or likely due to differences in experimental manipulation. COCs are matured in the presence of IVM hormones (LH, FSH, and estradiol) where cumulus granulosa cells maintain communication with the oocyte; thus, COCs that are examined experience changes associated with the maturation period. The granulosa cells that were cultured separately were removed from the oocyte prior to maturation and likely represent an alternate stage of cumulus granulosa cell development with pre-maturation expression patterns. This is taken into account using a control group that allows comparisons between groups but not amongst sample types. For this reason, this study does not aim to compare granulosa cells and COCs, but rather, reports the findings of two separate experimental models and accounts for these combined observations when interpreting the data.

Concisely, the significance of these finding consists in the understanding of the role of a key microRNA in early female reproduction, miR-21, and how it is affected by Endocrine Disrupting Compounds, such as Bisphenol A (BPA), ultimately affecting fertility. BPA has been proven to have deleterious effects in early reproduction as it is widely spread in the environment, eliciting its action at low doses of exposure. Although extensively studied, BPA mechanism of action and impacts on molecular aspects of granulosa cells viability remains elusive, especially at the non-genomic and epigenetic level. miR-21 is a well-studied microRNA that is proven as a crucial miRNA in regulating DNA methylation; it is also the most documented miRNA to be affected by BPA. This study investigates the involvement of miR-21 in BPA-induced aberrant DNA methylation and characterizes its potential molecular mechanism of action. This significantly contributes to the field of molecular biosciences particularly focused on epigenetic modulation of a specific microRNA by environmental toxicants. Countless miRNAs are continuously being identified on lengthy lists as differentially expressed in different physiological and pathological conditions. It is crucial to unpack these findings and work up with a single miRNA, tracking its mechanism to better understand its function. This may ultimately help us to further regulate BPA exposure and to better understand its role in female infertility.

## 4 Materials and methods

### 4.1 Cumulus oocyte complex (COC) collection, *in vitro* maturation, and COC transfections

The anti-miR-21 Locked Nucleic Acid (LNA) and the nonspecific scrambled control LNA were purchased from Qiagen (Toronto, ON, Canada). The LNA sequence was complementary to miR-21: 5′-CAA​CAT​CAG​TCT​GAT​AAG​CT-3′, and the scrambled control was a random mix of nucleotides: 5′-TAA​CAC​GTC​TAT​ACG​CCC​A-3′.

Bovine ovaries (*Bos taurus*) were collected from a local abattoir (Cargill Meat Solutions, Guelph, ON, Canada). Ovaries were transported at a temperature between 34–36°C and washed with sterile saline solution. COCs were aspirated from follicles using a vacuum pump and collected into a tube containing 1 mL of oocyte collection media comprised of 1 M HEPES-buffered Ham’s F-10 media (Sigma-Aldrich) supplemented with 2% Fetal Bovine Serum (Gibco), Heparin (0.2 IU/mL) (Fresenius Kabi Canada Ltd.), Sodium Bicarbonate (Sigma), and Penicillin/Streptomycin (1%) (Gibco). COCs were divided into 6 groups of 20 COCs per group into *in vitro* HEPES buffered TCM199 (Sigma-Aldrich) maturation media (S-IVM) containing 20% steer serum and sodium pyruvate (Sigma-Aldrich) and supplemented with 1 μg/mL LH (NIH), 0.5 μg/mL FSH (Follitropin V), 1 μg/mL Estradiol (Sigma-Aldrich).

This initial pilot experiment was to optimize miR-21 knockdown conditions in COCs and compare transfection efficiencies at different concentrations in the presence and absence of a transfection reagent (Lipofectamine 3,000; ThermoFisher). Transfection reagents are commonly used and are necessary to deliver the LNAs into most cell types but are cytotoxic and can introduce confounding results. The inhibitors purchased in this study have the potential to enter cells without the use of a transfection reagent if used at higher concentrations. To determine the most optimal conditions for our cell type and our experimental setting, it is crucial to assess the efficiency of knockdown with and without lipofectamine. The 6 groups were prepared as follows: Control group with S-IVM only, Lipofectamine alone in S-IVM (Vehicle), anti-miR-21 LNA in Lipofectamine in S-IVM (0.1 μM), Scramble LNA in Lipofectamine in S-IVM (0.1 μM), anti-miR-21 LNA alone in S-IVM (0.5 μM), and Scramble LNA alone in S-IVM (0.5 μM). Higher concentrations are needed for unassisted uptake while lower concentrations were used with lipofectamine as per the manufacturer’s recommendations. Samples were matured for 24 h at 38.5°C and 5% CO_2_. After maturation, COCs were snap frozen in liquid nitrogen for miR-21 quantification using qPCR.

Once the transfection conditions were determined using qPCR, COCs were collected again, but this time divided into three groups of 60 COCs per group. The 60 COCs were placed into 4-well dishes with 20 COCs per well in 3 wells for a total of 180 COCs across the three groups. The three groups were the nontransfected COCs (S-IVM only), Scramble COCs (0.5 μM of scramble LNA in S-IVM), and the miR-21 knockdown group (0.5 μM of the miR-21 inhibitor LNA in S-IVM). COCs were incubated at 38.5°C and 5% CO_2_. Halfway through maturation at 12 h, each 4-well dish was further divided into 3 groups labelled “Control, Vehicle, and BPA” to result in 9 groups altogether. The following treatments were prepared as followed and were then added into their respective wells: S-IVM only (Control), 0.1% ethanol in S-IVM (Vehicle), and 0.05 mg/mL of BPA (Sigma-Aldrich) dissolved in 0.1% ethanol in S-IVM. COCs were put back into the incubator to complete maturation for another 12 h in a humidified atmosphere at 38.5°C and 5% CO_2_. After 24 h, 20 COCs per group for 9 groups were imaged to observe qualitative effects alone. The COCs were then frozen in liquid nitrogen and stored at −80°C for downstream RNA and protein analysis.

### 4.2 Granulosa cell culture

COCs were aspirated from follicles using the methods described above. Approximately 100–200 COCs were stripped of their granulosa cells using mechanical disruptions via a micropipette. Granulosa cells were placed in 15 mL conical tubes containing 8 mL of 1X Dulbecco’s Modified Eagle Medium (DMEM) (Gibco), glutamine (2 mM) (Sigma-Aldrich), and penicillin/streptomycin (1%). Cells were resuspended in DMEM supplemented with 20% FBS and cultured at 38.5°C in 5% CO_2_ for 6–7 days with media replacement every 48 h until no empty patches were observed. At 100% confluency, the cells were passaged twice, split at passage 2 into 9 different groups in DMEM containing serum (10% FBS) in 6 well plates at 1 × 10^5^ cells/mL. After 24 h, cells were serum restricted using OptiMEM for another 24 h before being transfected with the LNAs. Optimal GC transfection with these LNAs have been previously optimized as described in Sabry et al. (2022). Briefly, cells were transfected with 0.5 μM of both LNAs for 12 h. Transfected cells were then treated with a vehicle (0.1% ethanol) or BPA (0.05 mg/mL in 0.1% ethanol) in OptiMEM for 12 h then frozen in liquid nitrogen and stored at −80°C for RNA and protein analysis.

### 4.3 Global Methylation Assessment by 5 mC and 5 hmC quantification

Global methylation patterns were assessed by quantifying 5mC and 5 hmC using immunofluorescence followed by confocal microscopy and flow cytometry for COCs and granulosa cells (GCs), respectively. In both cases, COCs and GCs were matured and cultured under the conditions previously described. However, instead of snap freezing in liquid nitrogen, the cells were fixed in 4% paraformaledhyde (PFA) for 30 min at 37°C for GCs and for 1 h at Room Temp (RT) for COCs. Cells were then washed 2X in PBS before being permeabilized in 0.1% Triton X-100 with 5% BSA for 1 h at RT. Primary antibodies were prepared in the permeabilization buffer at 1:100 for both 5 mC (ab10805) and 5 hmC (ab214728) antibodies. Cells were then incubated in the primary antibodies for 1 h at RT and then washed 3X in PBS to remove unbound primary antibodies, followed by incubations in the alexa-fluor 488 conjugated secondary antibodies [Anti-Mouse for 5 mC (ab150113) and Anti-Rabbit for 5 hmC (ab150077)] for 45 min at RT in the dark. Negative controls were included for each antibody and each sample type by replacing the primary antibody with an Isotype control. The Mouse IgG1 Isotype (ab170190) was used for 5 mC and the Rabbit IgG Isotype (ab172730) was used for 5 hmC as negative controls. Following incubation in the secondary antibodies, samples were washed 3X in PBS.

GCs were then resuspended in PBS with 5% BSA, strained through 40 μM cell strainers, and run through the BD Accuri C6 Flow Cytometer to quantify the fluorescence, which was analyzed using FlowJo V10 on a minimum of 3 biological replicates. COCs were mounted onto slides and mixed with Dakocytomation. Slides were then sealed and visualized using an Olympus FV1200 Confocal Microscope. Images were then analyzed using ImageJ and corrected total cell fluorescence was calculated according to the following formula: [CTCF = Integrated Density—(Area of Selected cell X Mean Area of Background Fluorescence)]. This analysis was performed on 15 individual COCs per group for 9 groups resulting in 135 COCs analyzed for 5mC and 135 COCs for 5 hmC.

### 4.4 RNA isolation and cDNA synthesis

Total RNA was isolated using the miRNeasy Micro Kit (Qiagen, Toronto, ON, Canada) according to the manufacturer’s instructions from a minimum of three biological replicates for both COCs and GCs. RNA concentration and quality were measured using the Nanodrop 2000c (ThermoFisher). 1 μg of mRNAs and 0.2 μg of miRNAs were reverse transcribed using qScript complementary DNA (cDNA) Supermix (Quantabio, Beverly Hills, MA, United States) and miRCURY LNA RT kit (Qiagen), respectively, in a T100 Thermal Cycler (BioRad, Mississauga, ON, Canada). cDNA was diluted with RNase-free water to a concentration of 5 ng/μL for mRNAs and the miRNAs were diluted 1:60 as per manufacturer guidelines for qPCR amplification.

### 4.5 Quantitative PCR (qPCR)

mRNA and miRNAs expression levels of a minimum of three biological replicates were quantified via quantitative real-time PCR (qPCR) using a CFX96 Touch Real-Time PCR Detection System (BioRad). mRNA was amplified using the SsoFast EvaGreen Supermix (BioRad), while miRNAs were amplified using the miRCURY LNA SYBR Green PCR Kit (Qiagen). Primers for mRNAs were purchased from Sigma-Aldrich and primers for miRNAs were purchased from Qiagen as predesigned primers from the miRCURY LNA miRNA PCR Assays. All primers were tested using standard curves with efficiencies accepted only with values between 90% and 110%. Gene expression was calculated using the efficiency-corrected method (ΔΔCt). Primer sequences and efficiencies can be found in Table 1. mRNA expression was normalized to housekeeping genes *Glyceraldehyde 3-phosphate dehydrogenase (GAPDH)*, *Tyrosine 3-monooxygenase/tryptophan 5-monooxygenase activation protein zeta (YWHAZ)*, and *Beta-actin (ACTB)*, as they were determined to be the most stable reference genes according to a GeNorm Analysis using the CFX Maestro Software 2.3 (Sabry et al., 2022). miRNA expression was normalized to miR-191 and miR-106a, as they are stable reference targets across treatments. Quantification was run on at least three biological replicates in technical triplicates.

**TABLE 1:** microRNA and mRNA Primer Sequences used for qPCR.

MicroRNA	Primer ID	Accession #		Sequence (5′-3′)	E (%)	Source
miR-191	hsa-miR-191-5p	MIMAT0000440		CAA​CGG​AAU​CCC​AAA​AGC​AGC​UG	105.1	Qiagen^a^
miR-106a	hsa-miR-106a-5p	MIMAT0000103		AAA​AGU​GCU​UAC​AGU​GCA​GGU​AG	100	
miR-21	hsa-miR-21-5p	MIMAT0000076		UAG​CUU​AUC​AGA​CUG​AUG​UUG​A	100.2	

Gene Symbol	Gene Name	Product size (bp)	Accession #	Primer Sequence sets (5′ -3′)	E (%)	Source
YWHAZ	*Tyrosine 3- monooxygenase/tryptophan 5-monooxygenase activation protein zeta*	120	NM_174814.2	F: GCA​TCC​CAC​AGA​CTA​TTT​CC	100.3	Sharma and Madan (2022)
				R: GCA​AAG​ACA​ATG​ACA​GAC​CA		
ACTB	*Beta-actin*	186	NM_173979.3	F: CCT​TCC​TGG​GCA​TGG​AAT​CCT	97	Tscherner et al. (2018)
				R: TCT​TCA​TTG​TGC​TGG​GTG​CC		
GAPDH	*Glyceraldehyde-3-phosphate dehydrogenase*	153	NM_001034034.2	F: TTC​CTG​GTA​CGA​CAA​TGA​ATT​TG	99.8	Ferris et al. (2016)
				R: GGAGATGGGGCAGGACTC		
DNMT1	*DNA methyltransferase 1*	136	NM_182651	F: TTA​GCA​CCT​CAT​TTG​CCG​AGT​A	99.4	Misirlioglu et al. (2006)
				R: TAG​GTG​GAG​TCA​GGG​TTG​CTC​T		
DNMT3A	*DNA methyltransferase 3A*	110	NM001206502.1	F: GCG​TTA​GTG​ACA​AGA​GGG​ACA	99.5	O’doherty et al. (2012)
				R: AAG​GTT​CCC​CCA​GAA​GTA​GC		
DNMT3B	*DNA methyltransferase 3B*	103	NM181813.2	F: GAA​ACC​AGG​ACT​CGG​TCT​GA	100.3	
				R: GGC​CTC​GGG​TAG​AAC​GTA​G		
TET1	*Ten-eleven translocation methylcytosine dioxygenase 1*	214	XM_003587999.2	F: CAA​AAC​CAG​GTG​GCG​CTT​G	100.1	Gao et al. (2020)
				R: GCA​GGC​TCT​GTT​TTA​TTT​CCA​C		
TET2	*Ten-eleven translocation methylcytosine dioxygenase 2*	285	XM_005207682.1	F: AAG​GCT​GAG​GGA​CGA​GAA​CGA	100.1	Zhang et al. (2020)
				R: GAG​ACG​GAG​ATG​GTA​TCA​AGA​ATG​G		
TET3	*Ten-eleven translocation methylcytosine dioxygenase 3*	118	XM_005212473.1	F: TCC​TTC​GGT​TGT​TCC​TGG​AG	96.9	
				R: TCT​TCC​GGA​GCA​CTT​CTT​CC		
TDG	*Thymine DNA Glycosylase*	159	NM_001083696.2	F: GAA​CGC​GGG​CAG​CTA​TTC​TC	101.4	Designed and sequenced (Supplementary Figure S1)
				R: GTC​TCT​CGT​GTG​GGT​TCC​TG		

^a^ miRNA primers were predesigned by Qiagen as part of a closed PCR system called miRCURY LNA PCR assays that contain sequences validated by the manufacturers and tested for efficiencies in this study.

miRNA PCR signal acquisition was carried out using the following two-step PCR cycling protocol: 95°C for 2 min followed by 40 cycles of 95°C for 10 s and 56°C for 60 s, ending with melt curve acquisition from 60°C to 95°C. mRNA PCR signal acquisition was carried out using the following two-step PCR cycling protocol: 95°C for 2 min followed by 44 cycles of 95°C for 10 s, 60°C for 30 s, ending with melt curve acquisition from 60°C to 95°C.

### 4.6 Protein isolation and western blot analysis

All Western blotting buffers and reagents were made in-house unless otherwise specified.

Quantification of DNMT1, DNMT3A, and TET2 proteins were performed by Western blotting on a minimum of 3 biological replicates in both GCs and COCs. Samples were lysed in 50 μL radioimmunoprecipitation assay (RIPA) buffer and 1% (*v*/*v*) protease inhibitors (Biotool, Jupiter, FL, USA), followed by freeze–thaw cycles in liquid nitrogen. Samples were then sheared using 0.33 mm (29 G) syringes (BD Biosciences) to break down the clumping of genomic DNA, placed in a water bath sonicator for 30 min followed by centrifugation at 12,000 × *g* RPM at 4°C for 10 min. Protein concentrations were quantified using the Bio-Rad DC protein assay (BioRad) and 40 and 30 μg of proteins for GCs and COCs, respectively, were loaded onto gels. Equal volumes of 3 ×reducing buffer with β-mercaptoethanol (Sigma-Aldrich) were added to each sample. Polyacrylamide gels (8%) were prepared using Bio-Rad standard gel recipes.

Proteins were heated for denaturation, separated on the 8% gels in an Invitrogen wet transfer Western blot apparatus (Invitrogen, Burlington, ON, Canada) at 125 V for 2 h and then transferred (40 V for 2 h) onto nitrocellulose membranes (Biorad) using a transfer buffer of Tris, Glycine, and water. Nitrocellulose blots were washed in Tris-buffered saline pH 7.6 with 0.1% Tween 20 (Thermo Fisher Scientific, Whitby, ON, Canada) (TBST), blocked for 1 h in 5% Bovine Serum Albumin (BSA) (Sigma-Aldrich) for TET2 and 5% Skim milk for DNMT1 and DNMT3A in TBST, to limit nonspecific binding. Blots were then incubated with each target primary antibody at 4°C overnight: DNMT1 at 1:500 (Cell Signaling D63A6, mAb#5032), DNMT3A at 1:1000 (ab228691), and TET2 at 1:500 (Cell Signaling, #45010).

After TBST washes, blots were incubated with the anti-rabbit IgG HRP-linked secondary antibody (Cell signaling Technology; 70,745) at 1:2000 dilution for DNMT1, 1:5000 for DNMT3A, and 1:1000 for TET2. All secondary antibodies were left on blots for 1 h at room temp and incubated with Clarity Western ECL Blotting Substrate (Bio-Rad) for 2–3 min. Blots were imaged on a ChemiDoc XRS + Imaging System (Bio-Rad). α-tubulin (Cell Signalling Technology) was used as a loading control and densitometric analysis was performed using the Bio-Rad Image Lab software and quantified as a ratio to α-tubulin expression.

### 4.7 Statistical analysis

GraphPad Prism 6 software was used to analyze the statistical difference among the treatment groups. Each data set was tested for normality using the Shapiro Wilk test for Normality. Normally distributed data sets were analyzed using a one-way Analysis of Variance (ANOVA) and non-parametric distributed data sets were analyzed using the Kruskal–Wallis test. Differences at a two-tailed *p*-value ≤0.05 were considered statistically significant. Parametric tests, specifically a one-way ANOVA, were suitable for normally distributed data as this test assumes homogeneity of variances and as such is sensitive to deviations from normality. When these assumptions are met, parametric tests offer greater statistical power and precision. Furthermore, a normally distributed data set that was found to have significant differences between the means of our tested variables was then subjected to a Tukeys Post-hoc test to establish which specific group differences are statistically significant. It helps identify which pairs of groups have means that differ significantly from each other after considering all the groups simultaneously. These differences are reported in the results section and in the graphs are denoted as different letters.

Non-parametric tests, like the Kruskal–Wallis test, are used for non-normally distributed data or data that does not meet the assumptions of parametric tests. They do not rely on the same distributional assumptions, which makes them more robust for skewed data and can also be useful when working with small sample sizes. Any data set that did not comply with parametric assumptions and found to have significant differences between the mean were also subjected to Dunn’s Multiple comparison tests which serves to perform *post hoc* pairwise comparisons between multiple groups in the context of non-parametric data. The data shown represent the mean ± standard error of the mean (SEM) for the biological replicates and statistical differences were determined at a two-tailed *p*-value ≤0.05, therefore, any differences with *p* ≤ 0.05 were considered significant.

## Data Availability

The raw data supporting the conclusion of this article will be made available by the authors, without undue reservation.
