# Supplementary material for: The relationship between miR-21, DNA methylation, and bisphenol a in bovine COCs and granulosa cells
Source: Front Cell Dev Biol. 2023 Nov 15;11:1294541. doi: 10.3389/fcell.2023.1294541 (PMC10684922; doi:10.3389/fcell.2023.1294541)
Supplement: Supplementary file 1 [file DataSheet1.pdf]

frontiers

Supplementary Material

1 Supplementary Figures and Tables

Supplementary Table 1. Sequenced Primer product for the bovine TDG gene

Gene	Primer	Sequenced Product	Percent Identity
TDG	**F:**GAACGCGGGCAGCTATTCTC<br><br>**R:**GTCTCTCGTGTGGGTTCCTG	NNNNNNNNNNNNNNNNNNNTTTCCNTTCCNA CAAATGATGACTGAAGCTCCTGACATGGCA GTTATAAACGCACAAGAAATGCCAGCCGAAGTTC CTGCCCCAGCACCTGCTCAGGAACCCAC ACGAGAGACA	98%

Bos taurus thymine DNA glycosylase (TDG), mRNA

Sequence ID: NM_001083696.2 Length: 3198 Number of Matches: 1

Range 1: 195 to 307 GenBank Graphics ▼ Next Match ▲ Previous Match

Score	Expect	Identities	Gaps	Strand
202 bits(109)	4e-50	111/113(98%)	0/113(0%)	Plus/Plus

```
Query  1    TTTCCNTTCCNACAAATGATGACTGAAGCTCCTGACATGGCAGTTATAAACGCACAAGAA  60
            ||||| |||| |||||||||||||||||||||||||||||||||||||||||||||||||
Sbjct  195  TTTCCATTCCAACAAATGATGACTGAAGCTCCTGACATGGCAGTTATAAACGCACAAGAA  254

Query  61   ATGCCAGCCGAAGTTCCTGCCCCAGCACCTGCTCAGGAACCCACACGAGAGAC  113
            |||||||||||||||||||||||||||||||||||||||||||||||||||||
Sbjct  255  ATGCCAGCCGAAGTTCCTGCCCCAGCACCTGCTCAGGAACCCACACGAGAGAC  307
```

Bos taurus thymine-DNA glycosylase, mRNA (cDNA clone MGC:140185 IMAGE:8168686), complete cds

Sequence ID: BC134451.1 Length: 3199 Number of Matches: 1

Range 1: 196 to 308 GenBank Graphics ▼ Next Match ▲ Previous Match

Score	Expect	Identities	Gaps	Strand
202 bits(109)	4e-50	111/113(98%)	0/113(0%)	Plus/Plus

```
Query  1    TTTCCNTTCCNACAAATGATGACTGAAGCTCCTGACATGGCAGTTATAAACGCACAAGAA  60
            ||||| |||| |||||||||||||||||||||||||||||||||||||||||||||||||
Sbjct  196  TTTCCATTCCAACAAATGATGACTGAAGCTCCTGACATGGCAGTTATAAACGCACAAGAA  255

Query  61   ATGCCAGCCGAAGTTCCTGCCCCAGCACCTGCTCAGGAACCCACACGAGAGAC  113
            |||||||||||||||||||||||||||||||||||||||||||||||||||||
Sbjct  256  ATGCCAGCCGAAGTTCCTGCCCCAGCACCTGCTCAGGAACCCACACGAGAGAC  308
```

PREDICTED: Bos taurus thymine DNA glycosylase (TDG), transcript variant X1, mRNA

Sequence ID: XM_024991735.1 Length: 3354 Number of Matches: 1

Range 1: 363 to 475 GenBank Graphics ▼ Next Match ▲ Previous Match

Score	Expect	Identities	Gaps	Strand
202 bits(109)	4e-50	111/113(98%)	0/113(0%)	Plus/Plus

```
Query  1    TTTCCNTTCCNACAAATGATGACTGAAGCTCCTGACATGGCAGTTATAAACGCACAAGAA  60
            ||||| |||| |||||||||||||||||||||||||||||||||||||||||||||||||
Sbjct  363  TTTCCATTCCAACAAATGATGACTGAAGCTCCTGACATGGCAGTTATAAACGCACAAGAA  422

Query  61   ATGCCAGCCGAAGTTCCTGCCCCAGCACCTGCTCAGGAACCCACACGAGAGAC  113
            |||||||||||||||||||||||||||||||||||||||||||||||||||||
Sbjct  423  ATGCCAGCCGAAGTTCCTGCCCCAGCACCTGCTCAGGAACCCACACGAGAGAC  475
```

Supplementary Figure 1 – Nucleotide Blast Results from sequenced TDG product. The product sequence was run through NCBI nucleotide blast resulting in 3 matches with 98% identities to the bovine TDG transcript.

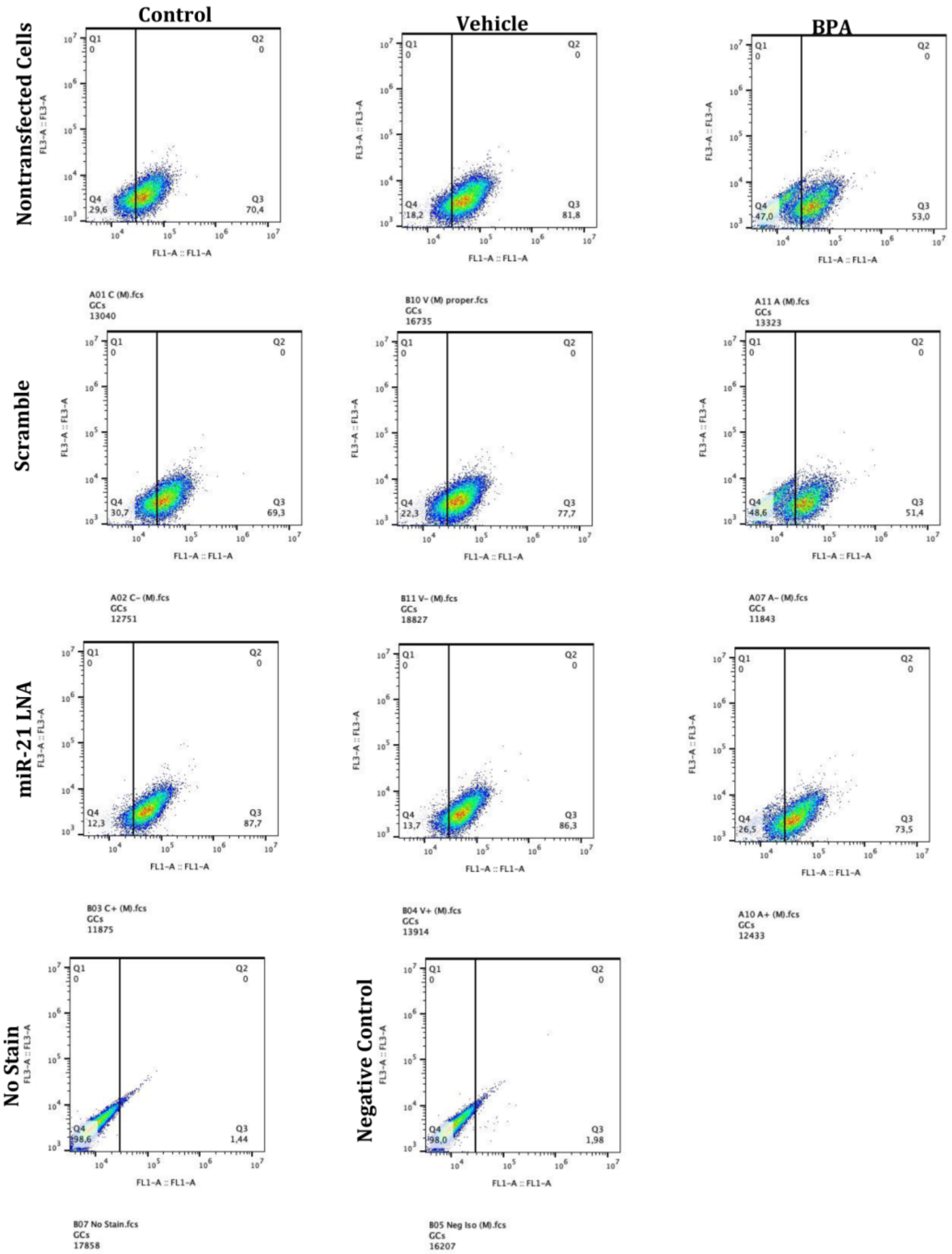

Supplementary Figure 2 – Flow cytometry of 5'methylcytosine (5mC) in Bovine Granulosa Cells. Transfected and treated GCs were fixed and permeabilized to allow for immunostaining of 5mC. Immunodetection of 5mC in GCs was achieved by utilizing the same Alexa Fluor 488 conjugated secondary antibody coupled with flow cytometry. Representative flow histograms are represented in for all 9 groups where the x-axis represents the FITC fluorescence from 5mC and the y-axis represents Hoechst stain from nuclear staining.

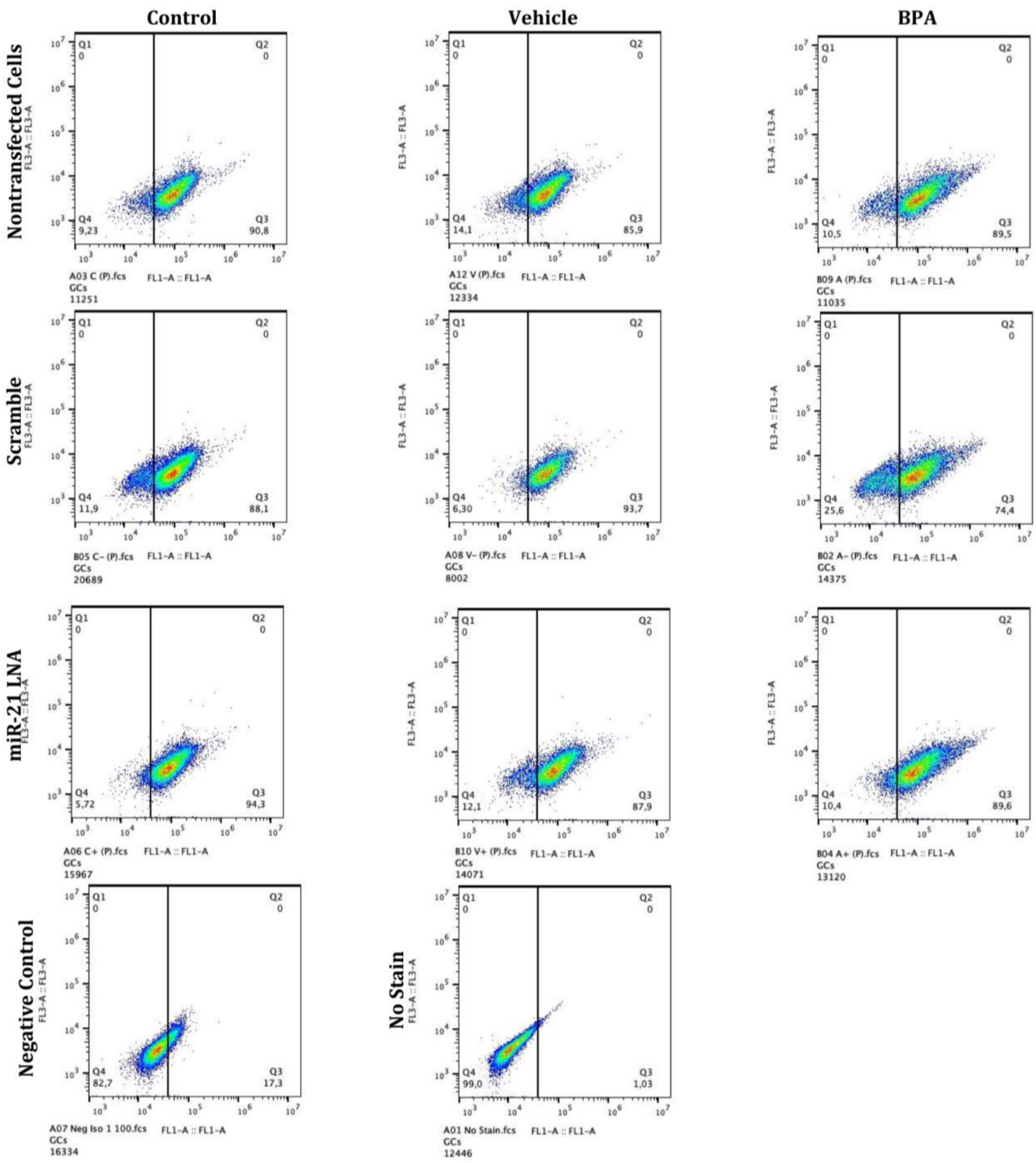

Supplementary Figure 3 – Figure 9. Flow cytometry of 5'hydroxymethylcytosine (5hmC) in Bovine Granulosa Cells. Transfected and treated GCs were fixed and permeabilized to allow for immunostaining of 5hmC. Immunodetection of 5hmC in GCs was achieved by utilizing the same Alexa Fluor 488 conjugated secondary antibody coupled with flow cytometry. Representative flow histograms are represented in for all 9 groups where the x-axis represents the FITC fluorescence from 5hmC and the y-axis represents Hoechst stain from nuclear staining.

Supplementary Table 2. Statistic reports from parametric and non-parametric analysis on mRNA data in COCs & GCs.

Gene	Normality	Statistic Report
**Cumulus Oocyte Complexes (COCs)**		
DNMT1	Skewed	[H(8)=20.0847, p=0.01002]
DNMT3A	Skewed	[H(8)=20.7973, p=0.007706]
TET1	Skewed	[H(8)=19.5026, p=0.01239]
TET2	Skewed	[H(8)=15.7989, p=0.04535]
TET3	Skewed	[H(8)=20.3919, p=0.008951]
TDG	Skewed	[H(8)=22.8694, p=0.003536]
**Granulosa Cells (GCs)**		
DNMT1	Skewed	[H(8)=20.4656, p=0.008711]
DNMT3A	Skewed	[H(8)=18.0175, p=0.0211]
TET1	Normal	[F(8,45)=3.840836, p=0.00162406]
TET2	Normal	[F(8,45)=2.51995, p=0.0234676]
TET3	Skewed	[H(8)=31.7993, p=0.0001012]
TDG	Skewed	[H(8)=30.0522, p=0.0002069]
